# Association of nocebo hyperalgesia and basic somatosensory characteristics in a large cohort

**DOI:** 10.1038/s41598-020-80386-y

**Published:** 2021-01-12

**Authors:** Mari Hanna Feldhaus, Björn Horing, Christian Sprenger, Christian Büchel

**Affiliations:** 1 Department of Systems Neuroscience, University Medical Center Hamburg-Eppendorf, Martinistr. 52, 20251 Hamburg, Germany; 2 Department of Anesthesiology, University Medical Center Hamburg-Eppendorf, Martinistr. 52, 20251 Hamburg, Germany

**Keywords:** Health care, Medical research, Psychology

## Abstract

Medical outcomes are strongly affected by placebo and nocebo effects. Prediction of who responds to such expectation effects has proven to be challenging. Most recent approaches to prediction have focused on placebo effects in the context of previous treatment experiences and expectancies, or personality traits. However, a recent model has suggested that basic somatosensory characteristics play an important role in expectation responses. Consequently, this study investigated not only the role of psychological variables, but also of basic somatosensory characteristics. In this study, 624 participants underwent a placebo and nocebo heat pain paradigm. Additionally, individual psychological and somatosensory characteristics were assessed. While no associations were identified for placebo responses, nocebo responses were associated with personality traits (e.g. neuroticism) and somatosensory characteristics (e.g. thermal pain threshold). Importantly, the associations between somatosensory characteristics and nocebo responses were among the strongest. This study shows that apart from personality traits, basic somatosensory characteristics play an important role in individual nocebo responses, in agreement with the novel idea that nocebo responses result from the integration of top-down expectation and bottom-up sensory information.

## Introduction

Pain is a multi-faceted phenomenon, with a large prevalence as a clinical symptom^1^, substantial impact on quality of life and high societal costs^2^. As with many subjective clinical symptoms, expectations substantially shape pain^3–6^*.* Implicit and explicit expectations can arise from sources as diverse as previous symptom experiences, verbal information or suggestions, or social observation^7^. In the case of pain, this has been demonstrated by effects on subjective ratings^8^, in pain-related neuronal changes in the brain and in the spinal cord^9,10^. Expectation-based positive treatment effects are commonly referred to as placebo effects, whereas negative effects (such as worsening of the outcome or the occurrence of side effects) are referred to as nocebo effects^7,11^.They substantially influence treatment success^12^ and the occurrence of adverse side effects^13–15^*.* Consequently, placebo and nocebo effects significantly shape patients’ everyday experiences with medical treatments making them an integral part of the treatment itself.

Even for established treatments, experiences based on conditioning processes as well as explicit expectations have been shown to substantially contribute to therapeutic outcome^13^. For example, the disclosure of possible side effects of a treatment already generates a nocebo expectation which can have negative consequences on the treatment outcome^16^. However, the impact of placebo and especially nocebo effects is ostensibly overlooked, particularly in clinical practice^8,17,18^. Consequently, studying the essential psychophysiological mechanisms and individual psychological and physiological foundations represents a major scientific and clinical objective. Moreover, the identification of key parameters determining placebo and nocebo responses can pave the way for individualized treatment optimization and minimizing expectation-based responses in clinical trials^8^.

Overarching concepts like the biopsychosocial model^19^ have been applied to expectation effects^20,21^ and emphasize the role of multiple classes of characteristics in their genesis. For example, the experience of pain would not only be affected by a patient’s illness itself and individual factors (e.g. personality, coping style), but also by physiological characteristics (e.g. nervous system makeup) and the social context (e.g. reinforcement or rejection). In the scope of this experimental study, both biological and psychological determinants were considered.

To date, possible associations of placebo and nocebo effects with individual characteristics have been investigated mainly in the psychological domain, especially with a focus on personality traits. For example, previous studies have identified dispositional optimism^22,23^, low state anxiety^23^, openness^24^, interoceptive awareness^24^, extraversion^23^, and reward sensitivity associated traits such as novelty seeking and behavioral drive^25^ as predictors for placebo effect responders. Regarding predictors for nocebo responses, a relationship with dispositional pessimism^22^, and neuroticism^26^ has been reported. In general, placebo effects have received far more attention than nocebo effects^11^, including recent studies involving large cohorts^27,28^. However, the importance of nocebo effects for quality of life, medication adherence and ultimately treatment success are being increasingly acknowledged^14,29^. In addition to personality traits, other features probably also play a role in the individual’s disposition for placebo and nocebo effects, such as cognitive factors like attention, which have been shown to interfere with pain processing^30,31^. In a novel framework, we highlighted the importance of the integration of expectation and sensory information to form a pain percept in the context of expectancy effects^32^. This notion has received conceptual^33^ and experimental support^34^ and implies that individual somatosensory characteristics such as pain thresholds also influence placebo and nocebo effects.

Based on these premises, we conducted an exploratory study investigating placebo and nocebo effects on pain, and their relation to individual characteristics. Individual characteristics included psychological traits captured through an extensive set of questionnaires, cognitive factors such as attention assessed by a working memory paradigm, and somatosensory characteristics quantified by quantitative sensory testing (QST). In order to identify relevant characteristics for placebo and nocebo effects, all characteristics were included in a variable selection analysis (LASSO, least absolute shrinkage and selection operator). The purpose of this study was to gain further insight into individual differences in placebo and nocebo effects by examining a large sample of healthy participants with a multi-faceted set of relevant characteristics.

## Results

### Placebo and Nocebo effects

**Figure 1:**
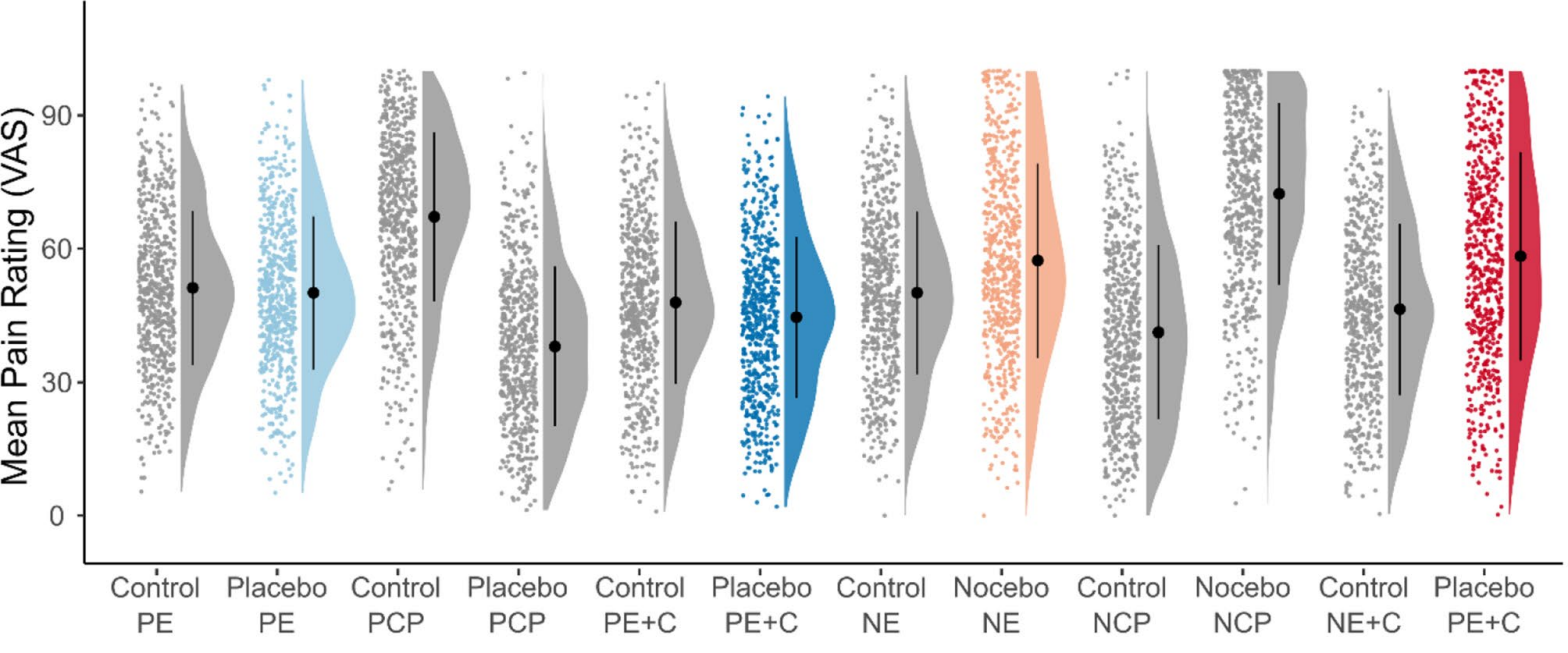
Mean pain rating per condition. The dots represent mean data (average of 8 trials) of individual participants. The half-violin plots represent the distribution of the data. The black dot represents the group mean and the error bars represent the standard deviation. The following conditions are displayed: Control condition rating Placebo Expectation (Control PE), placebo condition rating Placebo Expectation (Placebo PE), control condition rating Placebo Conditioning Procedure (Control PCP), placebo condition rating Placebo Conditioning Procedure (Placebo PCP), control condition rating Placebo Expectation plus Conditioning (Control PE + C), placebo condition rating Placebo Expectation plus Conditioning (Placebo PE + C), control condition rating Nocebo Expectation (Control NE), nocebo condition rating Nocebo Expectation (Nocebo NE), control condition rating Nocebo Conditioning Procedure (Control NCP), nocebo condition rating Nocebo Conditioning Procedure (Nocebo NCP), control condition rating Nocebo Expectation plus Conditioning (Control NE + C), nocebo condition rating Nocebo Expectation plus Conditioning (Nocebo NE + C). The sham treatment conditions used for calculation of the mean rating (against the respective control conditions) are displayed in color. In all conditions, participants were exposed to *VAS60* stimuli with the exception to the conditioning procedure. In the control condition of the Placebo Conditioning Procedure and the nocebo condition of the Nocebo Conditioning Procedure, the participant was exposed to *VAS80* stimuli, while in the placebo condition of the Placebo Conditioning Procedure and the control condition of the Nocebo Conditioning Procedure the participants were exposed to *VAS40* stimuli.

**Figure 2:**
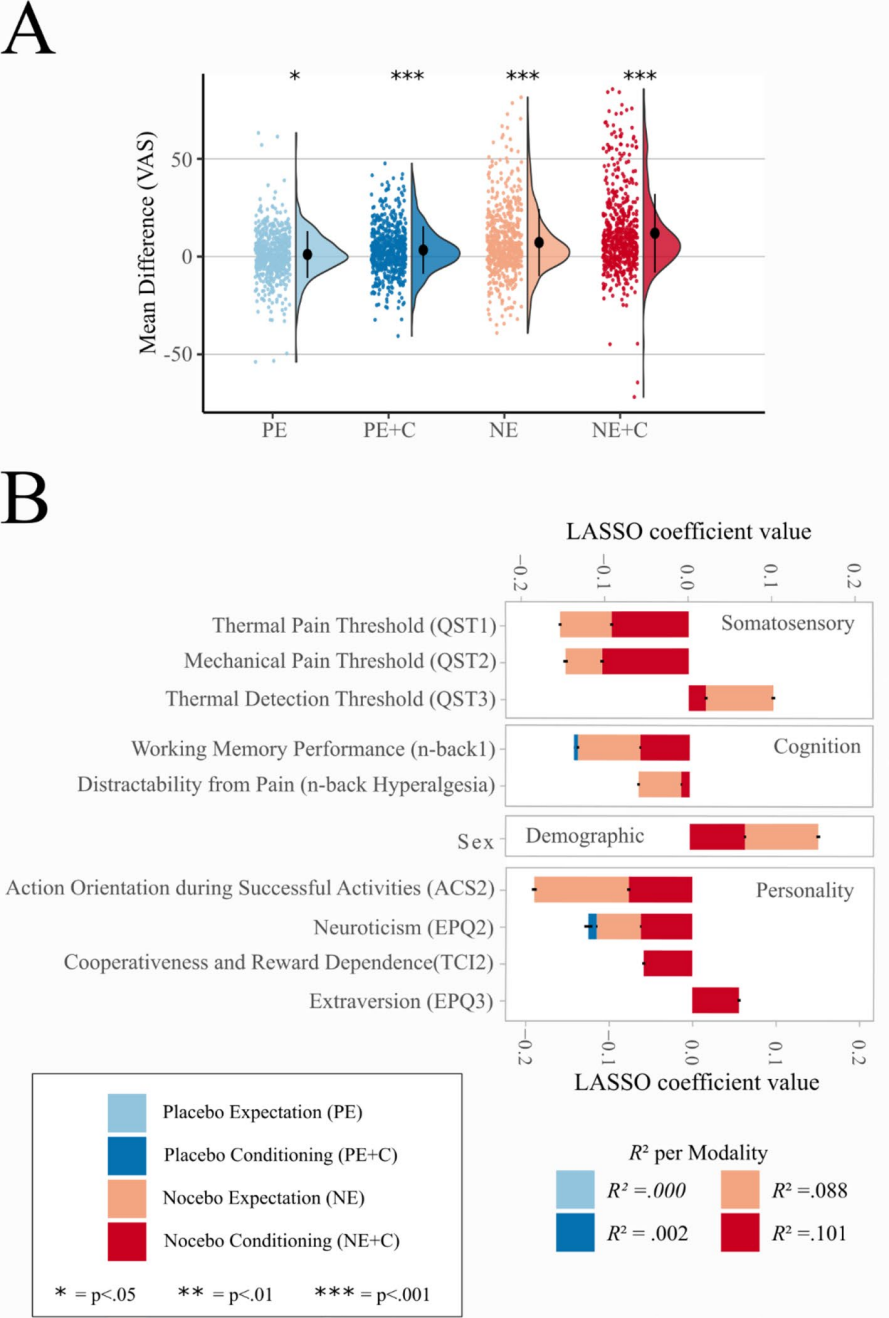
Main effects and LASSO analysis. (**A**) Group mean of placebo and nocebo effects (difference between sham treatment and control). The colored dots on the left represent raw jittered data of individual participants. The half-violin plots on the right represent the distribution of the data. The black dot represents the group mean and the error bars represent the standard deviation. The figure is color-coded for: Placebo Expectation (PE), Placebo Conditioning (PE + C), Nocebo Expectation (NE), Nocebo Conditioning (NE + C). (**B**) LASSO results. The stacked bar plots display the ten best LASSO coefficients for each modality with standard deviations of 1000 iterations. Variables are ordered by variable subgroup and within these subgroups ranked by summed overall coefficients.

This exploratory study aimed to distinguish individual characteristics that are associated with the extent of placebo and nocebo effects on painful heat. Placebo and nocebo responses were obtained from four consecutively applied experimental conditions involving expectancy and conditioning procedures for either modality. Figure 1 displays individual and mean pain ratings for all conditions. In a first step, we tested for placebo and nocebo responses using paired t-tests. The pairwise t-tests comparing sham treatment and control conditions revealed significant effects for placebo expectation (1.1% mean pain relief [95% CI, 0.1 to 2.0], *t*(610) = 2.20, *p* = 0.028, Cohen’s *d*_*z*_ = 0.09), placebo conditioning (3.3% mean pain relief [95% CI, 2.4 to 4.3], *t*(613) = 6.90, *p* < 0.001, Cohen’s *d*_*z*_ = 0.28), nocebo expectation (7.2% mean pain increase [95% CI, 5.9 to 8.6], *t*(608) = 10.65, *p* < 0.001, Cohen’s *d*_*z*_ = 0.43), and nocebo conditioning (11.9% mean pain increase [95% CI, 10.4 to 13.4], *t*(606) = 15.35, *p* < 0.001, Cohen’s *d*_*z*_ = 0.62). Figure 2A illustrates the main effects (Table 1).

**Table 1:** Group mean pain rating per condition. Group mean pain ratings and standard deviations for all conditions. Moreover, for each condition it is depicted to what rating the used temperature was calibrated to.

Condition	Group mean pain rating	Standard deviation	Temperature calibrated to
Control "Placebo Expectation" (Control PE)	51.17	17.29	*VAS60*
Placebo "Placebo Expectation" (Placebo PE)	50.07	17.20	*VAS60*
Control "Placebo Conditioning Procedure" (Control PCP)	67.16	19.06	*VAS80*
Placebo "Placebo Conditioning Procedure" (Placebo PCP)	38.06	17.91	*VAS40*
Control "Placebo Expectation plus Conditioning" (Control PC + E)	47.89	18.24	*VAS60*
Placebo "Placebo Expectation plus Conditioning" (Placebo PC + E)	44.58	18.04	*VAS60*
Control "Nocebo Expectation" (Control PE)	50.08	18.27	*VAS60*
Nocebo "Nocebo Expectation" (Nocebo PE)	57.32	21.84	*VAS60*
Control "Nocebo Conditioning Procedure" (Control PCP)	41.19	19.55	*VAS40*
Nocebo "Nocebo Conditioning Procedure" (Nocebo PCP)	72.35	20.45	*VAS80*
Control "Nocebo Expectation plus Conditioning" (Control PC + E)	46.37	19.27	*VAS60*
Nocebo "Nocebo Expectation plus Conditioning" (Nocebo PC + E)	58.30	23.45	*VAS60*

### Principal component analysis

To reduce the number of variables and increase interpretability, separate principal component analyses (PCA) for each assessment (e.g. questionnaire or QST) were performed. Principal components were characterized according to the subscales providing the highest loadings. An illustration of all individual loadings of the constructed principal components with the original variables can be found in Supplement Figure S1.

### Variable identification and regression analysis

In order to identify individual characteristics that are associated with placebo and nocebo effects, we used LASSO (least absolute shrinkage and selection operator) to perform both, a variable selection and regularization. LASSO was iterated 1000 times to ensure stable results and to estimate standard deviations of coefficients. Figure 2B illustrates the mean coefficients of the ten strongest predictors with standard deviations (see Supplement Figure S2 and Table S1 for all predictors). No associations with individual characteristics were determined for placebo expectation. For all other modalities, several predictors were identified: in placebo conditioning several principal components were selected inconsistently (in 35% of iterations), while in both nocebo modalities, several principal components were selected very consistently (in close to 100% of iterations; see Supplement Fig. S3 for detailed selection rates). For placebo conditioning 0.2% of the variance was explained (*R*^2^ = 0.002 [SD 0.003]). In contrast, for nocebo expectancy 8.8% of variance was explained (*R*^2^ = 0.088 [SD 0.002]) and for nocebo conditioning more than 10% of the variance was explained (*R*^2^ = 0.101 [SD 0.002]) by the model.

Regarding the LASSO coefficients, three of the six QST principal components showed strong associations with nocebo responses: Higher thermal pain thresholds (QST1) and higher mechanical pain thresholds (QST2) were associated with higher nocebo responses in both nocebo modalities. Furthermore, regarding nocebo expectation, lower detection thresholds (QST3) were associated with higher nocebo responses.

In addition to QST variables, sex was related to both nocebo modalities: Women showed larger nocebo responses than men. With regard to personality traits, higher action orientation during successful performance of activities (ACS2) was associated with higher nocebo responses in both nocebo modalities. Higher neuroticism scores (EPQ2) were also associated with higher nocebo responses in both nocebo modalities and with lower placebo responses in placebo conditioning. Moreover, higher cooperativeness and reward dependence (TCI2) and lower extraversion (EPQ3) were associated with higher nocebo responses in nocebo conditioning.

Working memory performance (n-back1) was associated with nocebo responses in both nocebo modalities, i.e. participants performing better in the more difficult task were more susceptible to nocebo responses. Moreover, higher distractibility from pain due to working memory load (n-back hypoalgesia) was associated with higher nocebo responses. For individual nocebo responses, control ratings and treatment ratings for the principal components with the ten highest LASSO coefficients, see Supplement Figure S4.

## Discussion

This large sample study investigated associations of a wide set of individual characteristics with placebo and nocebo responses in pain. We replicated associations between nocebo responses and certain previously reported personality traits such as neuroticism^26^, extraversion^35^ and reward processing^25^. Furthermore, we observed an association of distraction-induced hypoalgesia and nocebo effects. More importantly, we observed associations between nocebo effects and basic somatosensory traits as revealed by quantitative sensory testing. The latter result indicates that individual somatosensory characteristics are as important as psychological traits in determining expectation-dependent pain modulation.

Regarding the analysis of individual characteristics, PCAs were performed to decrease the number of variables. The principal components were interpreted based on their loading structure and are named accordingly in the following paragraphs*.* Associations of either expectancy effect with individual characteristics were selected through the LASSO variable selection analysis. They point to a scarcity of associations in the placebo conditions. However, several associations with individual characteristics were selected for both nocebo conditions (nocebo expectancy and nocebo expectancy plus conditioning). Most importantly, our data showed that basic sensory traits are associated with nocebo responses. Nocebo responses decreased with thermal detection thresholds (QST3), indicating that individuals with a more sensitive thermoception are more susceptible to negative expectations. A possible mechanism is attentiveness: Individuals with lower thermal detection threshold might have been more attentive during threshold estimation and therefore responded faster (yielding a lower threshold). The same individuals might have been also more attentive during nocebo instructions and therefore developed greater expectations and experienced greater nocebo effects.

Furthermore, nocebo responses were positively related to thermal pain thresholds (QST1) and mechanical pain thresholds (QST2) indicating that individuals with a higher pain threshold are influenced more strongly by negative treatment expectations. Under the assumption that individuals with a higher pain threshold need more nociceptive input to experience pain, the larger nocebo responses could be related to less precise processing of nociceptive information. A previously introduced Bayesian framework of how expectations can influence pain^32,33^ considers pain perception as the integration of prior knowledge (e.g. pain expectations and experiences) and incoming nociceptive information, which forms and updates the actual pain perception. According to this framework, the precision of nociception determines its relative contribution to actual pain perception: If the precision of nociceptive information is lower compared to the precision of the expectation (prior), the expectation gains more weight in the final perception. In other words: less precise nociception gives room for a stronger effect of expectations and thus a stronger nocebo response (see Supplement Fig. S5 for an illustration). Even though we exclusively investigated hyperalgesic effects, this finding suggests that unspecific side effects may also be associated with less precise nociception. Clinicians could hypothetically reduce potential side effects by assessing somatosensory characteristics in patients, and modifying their treatment strategy accordingly^36^. However, this hypothesis has to be further investigated. Furthermore, knowledge of these predictors would allow to minimize nocebo effects in randomized controlled trials, including side effects unrelated to the tested treatment, thereby increasing assay sensitivity^11,13^.

Research to identify associations of personality traits with placebo and nocebo responses reaches back decades^37^. However, it has been criticized based on whether context dependency allows unambiguous results^38^. Only replications across different contexts (e.g., experiments) allow to conclude some degree of context independence. Our data replicated several personality traits which have been associated with nocebo effects before, namely neuroticism^26^ and reward sensitivity^25^, making contextual effects less likely. In particular, our data shows that higher neuroticism (EPQ2) predicted nocebo responses, which matches the finding of Davis and colleagues^26^ that higher neuroticism scores are related to greater nocebo responses. We did not observe the previously reported association of placebo and nocebo responses with dispositional optimism^22,23^. Nevertheless, neuroticism is often moderately correlated with optimism^39,40^, which could explain why some studies identify optimism and others neuroticism as predictors. Moreover, an association of lower extraversion and conditioned nocebo responses was identified, which is consistent with other nocebo and placebo findings^35^.

Furthermore, nocebo responses were partially predicted by cooperativeness and reward dependence (TCI2), and action orientation during successful performance of activities (ACS2). The latter trait is related to self-regulation which has been identified as an important mechanism in expectation-based pain modulation^24^. Importantly, both traits are connected to reward: cooperativeness and reward dependence entail social reward, whereas action orientation during successful performance of activities describes being absorbed into an action and therefore the intrinsic reward of an action itself. These results support the notion by Schweinhardt and colleagues^25^, who postulated a relationship between placebo responsiveness and reward sensitivity associated personality traits.

The association of sex and nocebo responses was among the strongest, with women showing greater nocebo responses than men. This is in line with a meta-analysis covering six experimental nocebo studies^41^. However, it should be noted that in our study and in at least four out of six studies of the meta-analysis experimenters were all female. As individual pain experience is influenced by the experimenter’s sex^42^, future studies should further investigate the interaction of the participant’s and the experimenter’s sex on placebo and nocebo effects. In line with this and with our own results, a recent study demonstrated that participants reported more side effects if a person from the same gender was present^43^.

Nocebo responses were associated with cognitive load induced hypoalgesia: Participants who experienced less pain while involved in a demanding working memory task showed greater nocebo responses. While Buhle and colleagues^30^ found evidence for a dissociation of distraction and placebo expectation as pain modulators, our data suggests that at least for nocebo effects these two processes might be associated with each other. Nocebo responses were also predicted by better performances in the more difficult 2-back working memory task. Therefore, individuals who performed better and experienced less pain during the task, showed greater nocebo responses in the experiment. A possible mechanism is that these individuals might be generally more prone to externally initiated pain modulation.

The strength of this study is the large number of analyzed participants as well as the comprehensive set of characteristics that were investigated for associations with placebo and nocebo effects. However, this study also has several limitations. First, to prevent unnecessary variance (e.g. caused by diurnal variation) between participants, we used a fixed experimental order for all participants. Therefore, carry-over effects can develop, such as explicit or implicit assumptions concerning the intentions of the study. Judging from informal post-experimental interviews, there is no indication that this occurred, and participants did not question the alleged purpose of the experiment. Furthermore, a correlation analysis of placebo and nocebo effects revealed that placebo and nocebo effects were not correlated with each other (see supplement Fig S6 for an illustration of the correlation analysis). Second, the average placebo effects of 1 to 3 VAS points observed in this study are small. Nevertheless, the methodology employed here has repeatedly proven successful in inducing placebo effects in the past^9,34,44^. Moreover, other studies of comparable sample size have reported larger placebo effects^27,28^. It is therefore possible that the scarcity of measures associated with placebo responses was due to their small effect size, and the regression analysis for nocebo responses was more successful because of the higher effect sizes. Third, individual characteristics were assessed in between the placebo and nocebo paradigm. This ensured that participants were not exposed to too many subsequent pain stimulations. Whilst QST was performed on unaffected skin, it might have been methodologically advantageous to assess these characteristics at the start of the experiment. Fourth, the assumptions regarding the association of trait variables and outcomes are comparatively basic, and we did not consider more complex psychological process models recently applied to expectation effect research, such as the Elaboration Likelihood Model of persuasion^45^. Relatedly, expectations themselves were not assessed despite conceptual merit^6^, while empirical merit is more ambiguous^27,46^. Here, we opted against querying expectation ratings due to the risk of amplifying demand characteristics and carry-over effects inherent in a cross-over design^47^.

Nocebo mechanisms are an important cause for unspecific side effects. Therefore, it is very important to pinpoint individual characteristics that are associated with nocebo effects and help individualize treatment options. This large study replicated certain personality traits and more importantly, identified additional non-psychological, somatosensory characteristics as predictors for nocebo effects. Hence, conducting selective quantitative assessments of sensory systems in addition to more traditional psychological assessments could help to identify likely nocebo responders in order to improve accuracy in clinical trials and optimize patient treatment. Moreover, our results highlight the potential contribution of stable somatosensory characteristics to identify individuals susceptible to the nocebo effect—an endeavor that has mostly been pursued along the lines of psychological or genetic predictors^48^.

## Material and methods

### Study design

In this experimental study, a cross-over design was employed to test placebo and nocebo effects in the form of heat pain hypoalgesia or hyperalgesia. These primary outcomes were used to identify psychological and somatosensory characteristics. Data collection was performed at the University Medical Center Hamburg-Eppendorf. The study was approved by the Ethics Committee of the Medical Board Hamburg, Germany.

### Participants

The sample consisted of 720 healthy participants assessed exclusively for this project. The data was collected from March 2013 to September 2016. Participants gave written informed consent. All participants were fluent German speakers and reported no acute or chronic diseases, no pain medication intake during the last 24 h, and no damaged skin on either forearm. All participants underwent the same protocol (described in detail in the next paragraphs): They underwent an established placebo and nocebo paradigm^9,10^, filled out a set of questionnaires, completed a quantitative sensory testing (QST)^49^ and performed a working memory paradigm with co-occurring pain stimuli^31^. The experiment lasted for seven hours including a one-hour lunch break. Two participants were investigated at the same time supervised by the experimenter. Participants received €100 as a compensation for their attendance. All participants were debriefed after the experiment and given the opportunity to withdraw their data. No participant withdrew their data. The experiment was performed by two female study psychologists. Both received a 2-day formal training in QST. The first experimenter tested 310 participants, the second experimenter tested 410 participants. In all analyses, experimenter was used as a covariate to control for possible experimenter effects. As participants had to complete a high number of questionnaires and tasks at the computer, certain criteria were defined to identify careless responders that should be excluded. To identify these careless responders, the individual data was scanned for conspicuous patterns in the questionnaires^50^, tasks, and the placebo and nocebo paradigm (i.e. giving different answers to very similar questions or giving the same answer for more than 80 times in a row). These criteria led to the exclusion of 96 participants, resulting in a final sample of 624 participants (373 female, mean 24.6 [SD 3.6] years, range 18–35) who were included in the data analysis. For detailed exclusion criteria see Supplement Table S2. Detailed sample characteristics are provided in the Supplement Table S3.

### Pain stimulation

**Figure 3:**
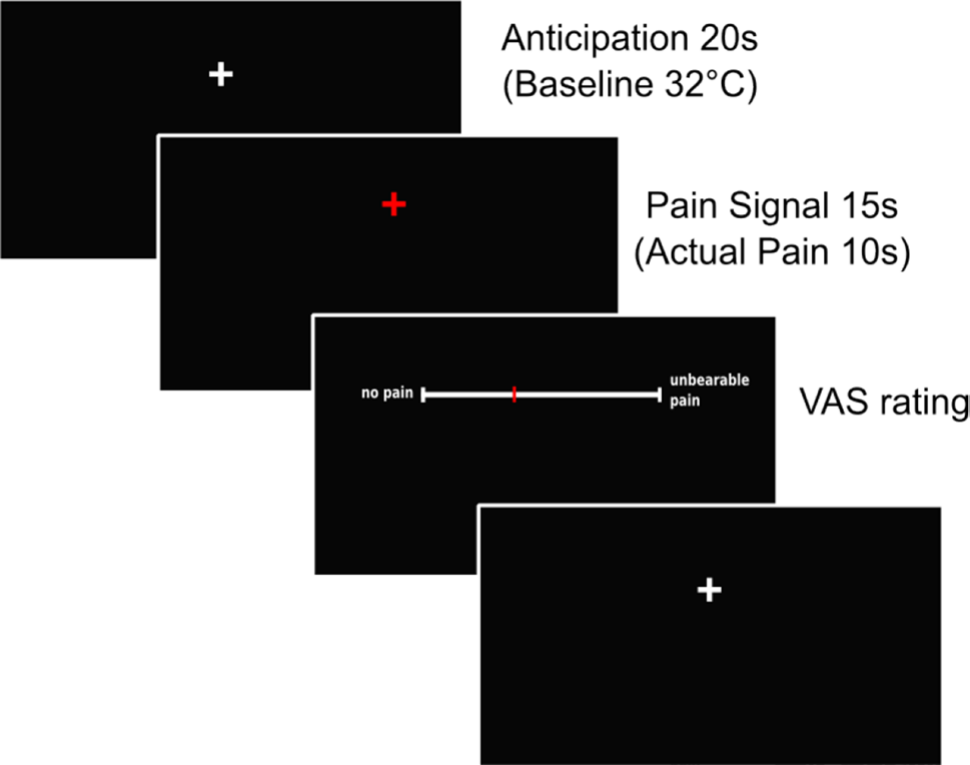
Pain stimulation during one experimental trial. Every experimental trial started with an anticipation phase of 20 s during which participants saw a white fixation cross. Afterwards, a red fixation cross appeared indicating the pain stimulation. The red fixation cross was shown for 15 s, while the actual pain stimulation lasted for 10 s. The pain stimulation was followed by the VAS pain rating.

Pain was evoked via a contact heat thermode (ATS-Thermode, Medoc LTD Advanced Medical Systems, Rimat Yishai, Israel). The contact area (30 × 30 mm) was attached to skin sites on the volar forearm. Baseline temperature was set to 32 °C and painful stimuli ranged from 42 to 48 °C. The heat stimuli duration was 10 s with a rise and fall rate of 8 °C/s. Figure 3 illustrates an experimental trial as it was used during calibration and test. At first, participants watched a black screen with a white cross. As soon as the white cross turned red, the heat stimuli began. Afterwards participants were asked to rate perceived pain on a visual analog scale (VAS) ranging from 0 (“no pain”) to 100 (“unbearable pain”) using keyboard arrow keys and confirming their rating with enter.

### Pain calibration

Heat levels were individually calibrated for each participant to achieve the same subjective aversive pain experience across participants. Temperatures were individually calibrated to match *VAS40* and *VAS80* using a stepwise procedure of 6 trials to approach a temperature which matched the chosen VAS rating. The mean of the *VAS40* and *VAS80* temperatures was used as the temperature for *VAS60* stimuli. The calibrated mean temperature (± SD) for *VAS40* was 44.8 ± 1.3 °C, for *VAS60* 45.7 ± 1.1 °C and for *VAS80* 46.7 ± 1.1 °C.

### Experimental protocol

**Figure 4:**
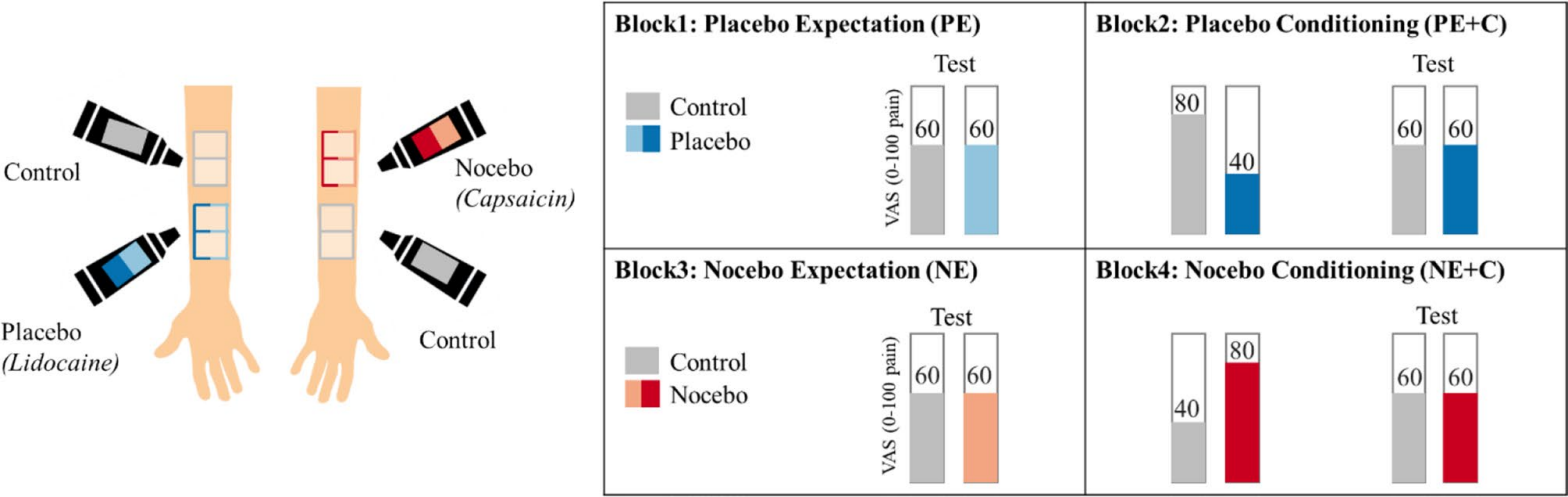
Placebo and nocebo paradigm. (**A**) Ointments and marked skin areas. For experimental block 1 and 2, a placebo ointment (“Lidocaine”) and a control ointment were introduced. It was explained that “Lidocaine” would exert an analgesic effect. Four skin areas were marked on one forearm of the participants. The skin areas were color-coded for “Lidocaine” and control treatment. For experimental block 1 and the conditioning part of experimental block 2, one control area and one placebo area were used, whereas the second phase (“test”) in experimental block 2 was performed on the remaining two skin areas on the same forearm. For experimental block 3 and 4, the nocebo ointment (“Capsaicin”) was introduced to the participants, as a treatment with a hyperalgesic effect. Four skin areas were marked on the other forearm. For experimental block 3 and the conditioning part experimental block 4, one control area and one nocebo area were used. Afterwards, the second phase (“test”) of experimental block 4 was performed at another nocebo area and control area. The order of the left and right arm and the position of placebo, nocebo and control were counterbalanced across participants. (**B**) Placebo and nocebo paradigm. In experimental block 1, placebo expectation (PE) effects were tested: Participants were exposed to two times eight heat stimuli, which were calibrated to match 60 on a 0 to 100 VAS (*VAS60*). In experimental block 2, placebo conditioning (PC) effects were tested. Therefore, participants were first conditioned (*VAS80* stimuli for control skin area and *VAS40* for placebo skin area) on the previous skin areas, and then tested again with *VAS60* on the remaining two skin areas. Afterwards participants were introduced to the nocebo treatment and experimental block 3 and 4 were performed in the same manner as experimental block 1 and 2, except that the nocebo skin area was conditioned with *VAS80* and the control skin area was conditioned with *VAS40*, respectively. The overall order of the blocks was fixed for all participants, while the order of treatment (placebo or nocebo) and control was counterbalanced across participants.

The following paragraphs will further explain the experimental protocol. The placebo and nocebo paradigm is illustrated in Fig. 4A and B.

#### Experimental introduction

Two participants arrived in the morning and were individually interviewed for any chronic or recent health issues, informed about the pain experiment and gave written informed consent. In the experimental room, participants were seated at two tables, separated by a room divider. The experimenter informed them about the alleged purpose of the study that was to examine associations of pain perception and personality, cognitive abilities, and skin sensitivity in the context of two different treatments. To further explain the two treatments, three different ointments were introduced. The first ointment was introduced as a lidocaine ointment that anesthetizes the skin and therefore decreases pain perception. The second ointment was introduced as a capsaicin ointment that irritates the skin and therefore increases pain perception. See Supplement Table S4 for the exact instructions. The third ointment was introduced as a control ointment that is neutral and has no effect on pain perception. Unbeknown to the participant, all ointments were identical and free of any active ingredient. The first ointment will further be called placebo ointment, the second ointment will further be called nocebo ointment and the third ointment will further be called control ointment. The participants were tested interleaved: When one participant was tested in the placebo- and nocebo paradigm, the other participant was answering questionnaires.

#### Block 1: Placebo expectation

As illustrated in Fig. 4A, four areas were marked on the inner forearm of the first participant. The placebo ointment was applied on two adjacent areas and the control ointment was applied on the other two areas. Left and right forearm as well as upper and lower forearm was randomized across participants. To give the ointment some time to “soak in”, temperatures were now calibrated on another skin area at the same forearm, as explained above. Now, the placebo effect based on pure expectation (by verbal suggestion) was assessed. The participant rated eight medium *VAS60* stimuli on placebo-treated skin area (placebo condition) and eight medium *VAS60* stimuli on a control skin area (control condition).

#### Quantitative sensory testing

Next, QST was performed according to the protocol of the German Research Network on Neuropathic Pain^49^ by QST-certified personnel. The following measures were assessed: cold detection threshold, warm detection threshold, cold pain threshold, heat pain threshold, mechanical detection threshold, mechanical pain threshold, dynamic mechanical allodynia, temporal pain summation (“Wind-up”), vibration detection threshold, and pressure pain threshold. For further details see the comprehensive protocol for clinical trials^49^.

#### Block 2: Placebo expectation plus conditioning

Now the second part of the placebo paradigm commenced. First, the conditioning procedure was performed: On the previously examined skin areas (in block 1), eight stimuli were applied and rated. Unbeknownst to the participant, the placebo-treated skin area was exposed to eight less painful *VAS40* stimuli to let them experience the effectiveness of the allegedly analgesic ointment, while the control skin area was exposed to eight more painful *VAS80* stimuli. Afterwards, the placebo effect was assessed using the remaining skin areas and applying eight *VAS60* stimuli on both, the placebo-treated and control-treated skin area (identical to block 1).

#### Block 3: Nocebo expectation

After a one-hour individual lunch break, participants were further introduced to the “pain enhancing” (nocebo) ointment. The nocebo ointment and the control ointment were applied to either two marked areas on the other forearm. The participants answered questionnaires while the ointment “soaked in” for five minutes. Afterwards, they were assessed for the nocebo effect based on pure expectation (by verbal suggestion). The participant rated eight medium *VAS60* stimuli on a nocebo-treated skin area (nocebo condition) and eight medium *VAS60* stimuli on a control skin area (control condition).

#### Working memory paradigm

**Figure 5:**
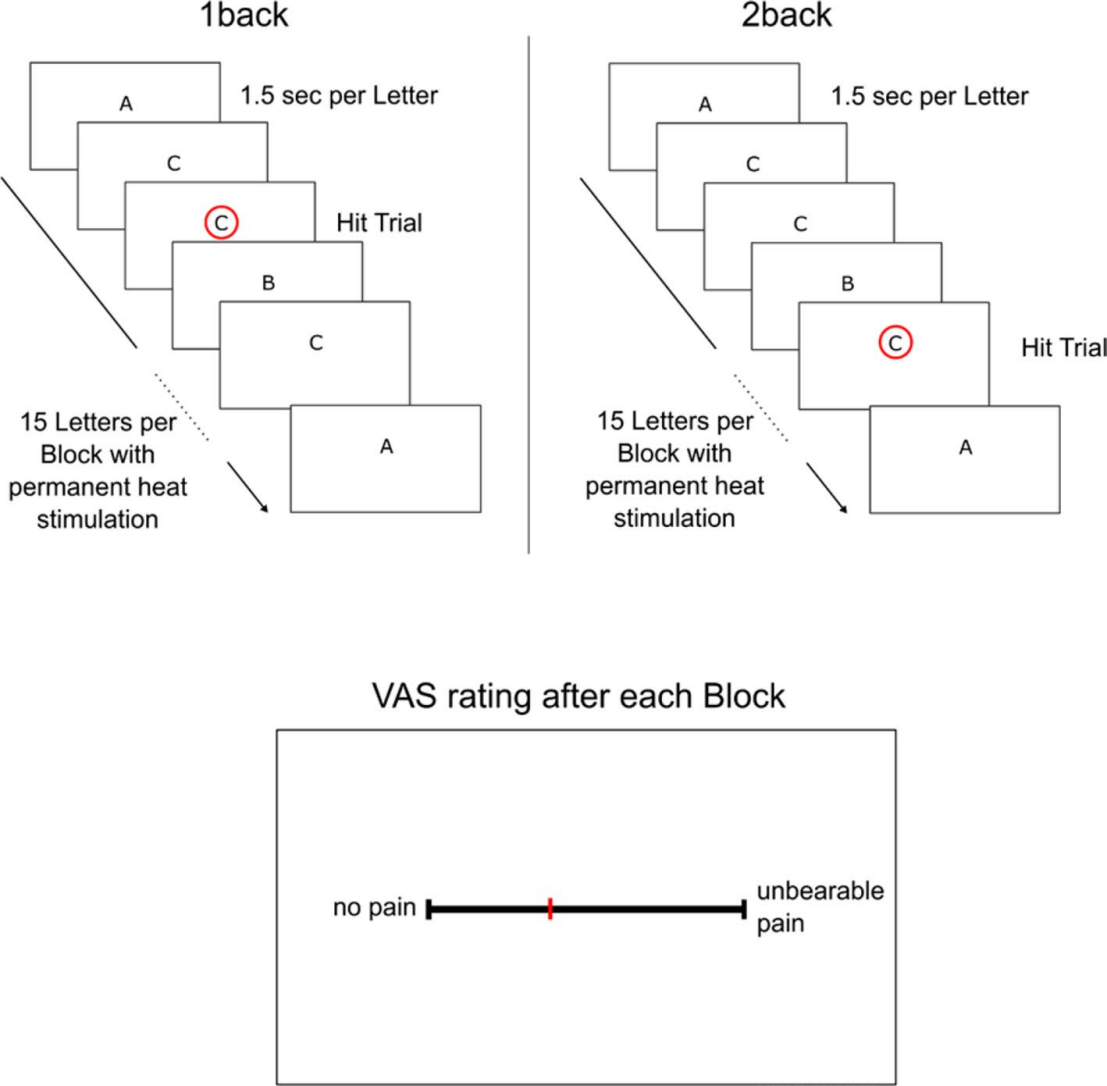
Working Memory Paradigm “n-back”. The upper left part illustrates the easier version of the working memory paradigm (“1-back”). The participant watched a stream of letters and had to respond when the same letter is presented two times in a row, as indicated by the red circle. The upper right part illustrates the more difficult version of the working memory paradigm (“2-back”). The participant watched a stream of letters and had to respond when the letter was the same as the one before the last letter, as indicated by the red circle. While the participant was involved in the task, a heat pain was applied on the forearm, and the participants rated their heat pain experience after each block on a visual analogue scale.

At some point during the alternating experimental tasks, either participant underwent the working memory paradigm. Working memory capacity and distraction induced hypoalgesia were assessed by a working memory paradigm using an n-back task^31^. The task is illustrated in Fig. 5. The participants’ task was to concentrate on a stream of letters, which were presented successively in the middle of the screen. For the basic level (“1-back”), the participant had to respond as fast as possible whenever one letter was the same as the letter shown before, as indicated with a red circle in Fig. 5 on the left side. For the advanced level (“2-back”), the participant had to react as fast as possible whenever the letter was the same as the one before the last letter, as indicated with a red circle in Fig. 5 on the right side. One block consisted of 15 successively presented letters. While the participant was involved in the task, a heat pain stimulus calibrated to match *VAS60* was applied at the participants forearm for each block and afterwards rated on a 0 to 100 VAS scale. Hit rates, false alarm rates, error rates, reaction times and VAS ratings were recorded. We analyzed the difference scores of “2-back” minus “1-back”, as this contrasts high working memory load to low working memory load.

#### Block 4: Nocebo expectation plus conditioning

Now the second part of the nocebo paradigm commenced. As in block 2, an additional conditioning was introduced: On the previously examined skin areas (in block 3), eight stimuli were applied and rated. Unbeknown to the participant who believed that the temperatures remained unchanged, the nocebo-treated skin area was exposed to eight more painful *VAS80* stimuli to let them experience the effectiveness of the allegedly sensitizing ointment, while the control skin area was exposed to eight *VAS40* stimuli. Afterwards, the nocebo effect was assessed using different skin areas and applying eight *VAS60* stimuli on both, the nocebo-treated and control-treated skin area (identical to block 3). The difference of these ratings was considered as the nocebo effect base on expectation plus conditioning. After the nocebo paradigm was completed participants were fully informed about the actual purpose of the study.

#### Questionnaires and additional secondary assessments

Questionnaires were answered whenever the participant was not directly involved in the placebo/nocebo paradigm, QST, or the working memory paradigm. The following questionnaires were assessed: Action Control Scale 90 (ACS-90), Anxiety Sensitivity-Index3 (ASI), Beliefs about Medicines Questionnaires (BMQ), Cognitive Emotion Regulation Questionnaire (CERQ), Center for Epidemiologic Studies Depression Scale (CES-D-Scale), Defensive Pessimism Questionnaire (DPQ), Emotion Regulation Questionnaire (ERQ), German Extended Personal Attributes Questionnaire (GEPAQ), General Competence Expectancy Test (GKE), Internality, Powerful Other and Chance Scale (IPC), Life-Orientation-Test (LOT), Pain Catastrophizing Scale (PCS), Pain Vigilance and Awareness Questionnaire (PVAQ), Symptom Checklist 90 (SCL 90), Social Desirability Scale-17 (SDS-17), State Trait Anxiety Inventory (STAI), Temperament Character Inventory (TCI). Additionally, sex, age, and body mass index (BMI) were assessed. See Supplement Table S5 for list of questionnaires with references. For all questionnaires, subscale scores were calculated according to the manual of the questionnaire, see Supplement Figure S1 and Supplement Table S3 for used sub-scales.

### Placebo and Nocebo effect estimation

For all conditions, the mean pain rating across the eight trials were calculated. Placebo and nocebo effects were defined as the difference in mean pain ratings between control ratings and (sham) treatment ratings. Placebo effects were defined as control ratings minus placebo ratings (for identical *VAS60* stimuli), while nocebo effects were defined as nocebo ratings minus control ratings, leading to positively coded effects in placebo and nocebo blocks. This within-participant design allowed for an estimation of placebo and nocebo effects for each participant, which enabled us to identify associations of placebo and nocebo effects with individual traits.

Placebo and nocebo effects are expectancy driven. These expectations can be shaped by verbal suggestions and conditioning, whereas conditioning seems to strengthen verbally suggested expectancy^51–53^. Consequently, we tested placebo and nocebo effects by pure expectation induced by verbal suggestions and by classical conditioning.

The order of the experimental blocks was identical for all participants, as the aim of this study was the identification of predictors for placebo and nocebo effects. Moreover, because of learning effects, the order of pure expectation effects and expectation effects plus conditioning could not be changed. Although a fixed order confounds the relative difference between placebo and nocebo effects with time of test (morning vs afternoon), it also reduces the variance across participants, and thus can increase the statistical power to identify associations with individual characteristics.

### Statistical analysis

To account for collinearity within domains and increase interpretability, measures were restructured using PCAs (R package “psych”, version 1.7.8). These were calculated for QST, the cognitive task and the individual questionnaires (using the questionnaire scales). The number of principal components in each domain was chosen as to explain at least 70% of the total variance. This procedure reduced the number of predictors from 75 to 46. All variables and components were rescaled and centered.

Our experimental aim was to identify associations of placebo and nocebo effects with individual characteristics. To prove a successful induction of placebo and nocebo effects, we tested the placebo and nocebo ratings effects using repeated measures ANOVA and paired sample t-tests.

To examine associations of individual characteristics with placebo and nocebo effects, a regularized regression analysis with variable selection (LASSO)^54^ was employed. LASSO minimizes the residual sum of squares by imposing a penalty (regularization) and therefore reduces correlated coefficient values towards zero^54^, which enhances the prediction accuracy and interpretability of the predictors. This penalty for correlated variables was beneficial to our set of principal components, as they could still be correlate given that the PCA was performed within each questionnaire, QST and working memory paradigm. For an intercorrelation analysis of the principal components see Supplement Figure S7. As a result, LASSO obtained a subset of the original characteristics which has the advantage of higher prediction accuracy and better interpretability compared to a non-regularized regression model^54^. LASSO was performed using the R-package “glmnet”, version 2.0-13. Missing data was imputed in each iteration, and results were cross-validated. Because of the imputation of missing data and the cross-validation, LASSO selection and coefficient estimation could differ depending on tuning parameters. Therefore, the analysis was repeated 1000 times for placebo and nocebo responses, which secured stable results. In all analyses, we controlled for experimenter and absolute calibrated temperature as covariates. All analyses were performed using R version 3.4.2 software.

### Ethical approval

The study was approved by the Ethics Committee of the Medical Board Hamburg, Germany (PV4030), and it was conducted in accordance with the World Medical Association Declaration of Helsinki. All participants provided verbal and written consent to participate.

## Supplementary information

Supplementary Information 1.
