# Supplementary material for: Association of nocebo hyperalgesia and basic somatosensory characteristics in a large cohort
Source: Sci Rep. 2021 Jan 12;11:762. doi: 10.1038/s41598-020-80386-y (PMC7804006; doi:10.1038/s41598-020-80386-y)
Supplement: Supplementary file 1 — Supplementary Information 1. [file 41598_2020_80386_MOESM1_ESM.docx]

***Supplementary materials***

***Association of nocebo hyperalgesia and basic somatosensory characteristics in a large cohort***

Mari Hanna Feldhaus^1^*, Björn Horing^1^, Christian Sprenger^1,2^, Christian Büchel^1^

^1^Department of Systems Neuroscience, University Medical Center Hamburg-Eppendorf, Hamburg, Germany

^2^Department of Anesthesiology, University Medical Center Hamburg-Eppendorf, Hamburg, Germany

*Corresponding Author: Mari Hanna Feldhaus, Department of Systems Neuroscience, University Medical Center Hamburg-Eppendorf, Martinistr. 52, Hamburg, 20251, Germany, E-mail: mari.feldhaus@gmx.de

The authors report no conflicts of interest.

# List of Abbreviations

| **ACS** | Action Control Scale 90 |
| --- | --- |
| **ASI** | Anxiety Sensitivity-Index-3 |
| **BMQ** | Beliefs about Medicines Questionnaires |
| **CERQ** | Cognitive Emotion Regulation Questionnaire |
| **CES-D** | Center for Epidemiologic Studies Depression Scale |
| **DPQ** | Defensive Pessimism Questionnaire |
| **EPQ** | Eysenck Personality Questionnaire |
| **GEPAQ** | German Extended Personal Attributes Questionnaire |
| **GKE** | General Competence Expectancy Test |
| **IPC** | Internality, Powerful Other and Chance Scale |
| **LASSO** | Least Absolute Shrinkage And Selection Operator |
| **LOT** | Life-Orientation-Test |
| **NCP** | Nocebo Conditioning Procedure |
| **NE** | Nocebo Expectation |
| **NE+C** | Nocebo Expectation Plus Conditioning |
| **PCP** | Placebo Conditioning Procedure |
| **PCS** | Pain Catastrophizing Scale |
| **PE** | Placebo Expectation |
| **PE+C** | Placebo Expectation Plus Conditioning |
| **PVAQ** | Pain Vigilance and Awareness Questionnaire |
| **QST** | Quantitative Sensory Testing |
| **SCL** | Symptom Checklist 90 |
| **SD** | Standard Deviation |
| **SDS** | Social Desirability Scale-17 |
| **STAI** | State-Trait Anxiety Inventory |
| **TCI** | Temperament Character Inventory |
| **VAS** | Visual Analogue Scale |


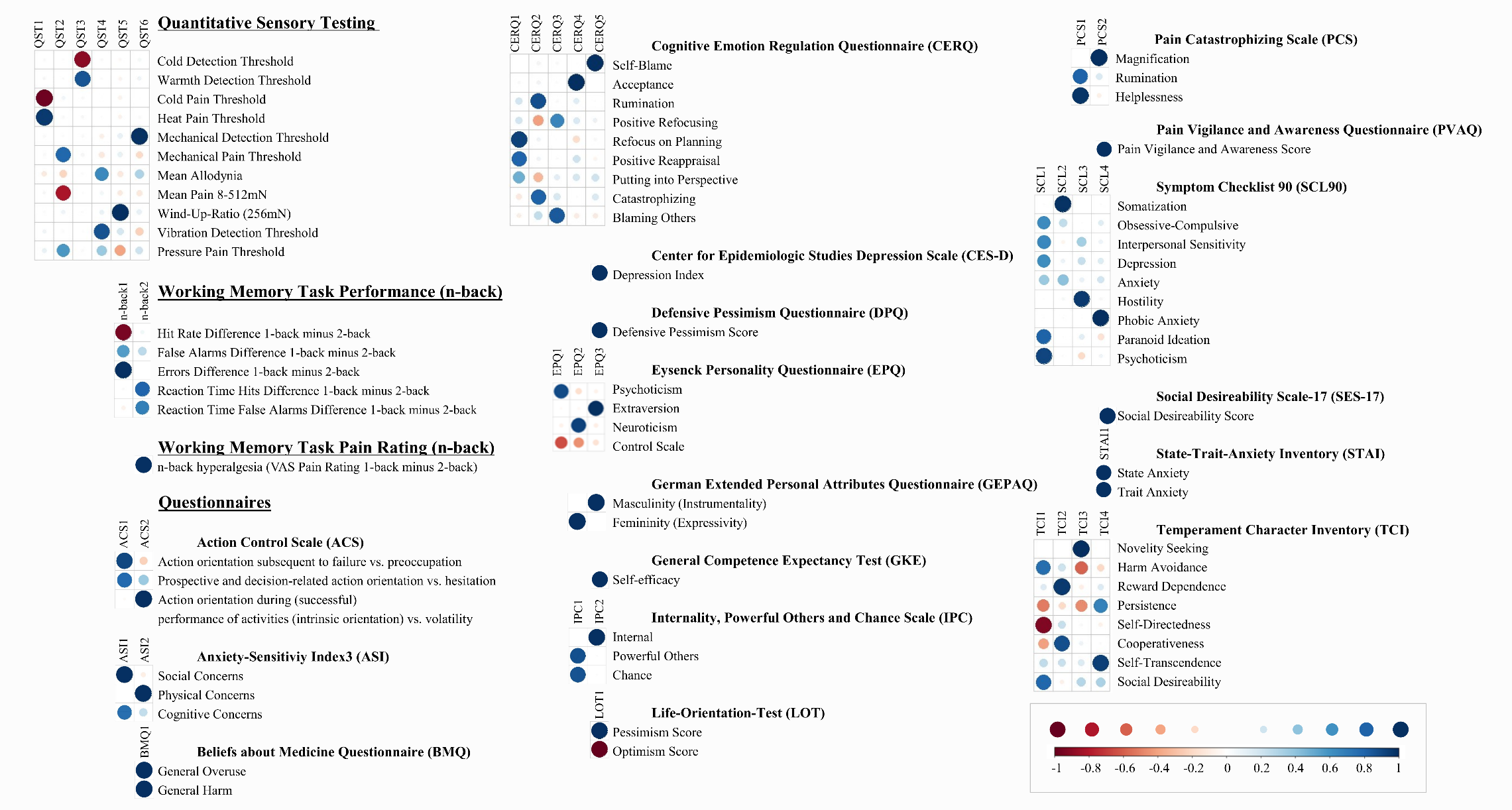
**Fig. S1: Loadings of individual characteristics on principal components.** For each questionnaire, working memory task and quantitative sensory test, PC analyses were calculated separately. Circle size reflects the loading of the individual variables with the corresponding principal component. Blue circles indicate positive loadings and red circles indicate negative loading.


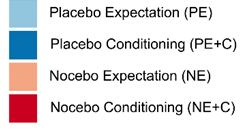

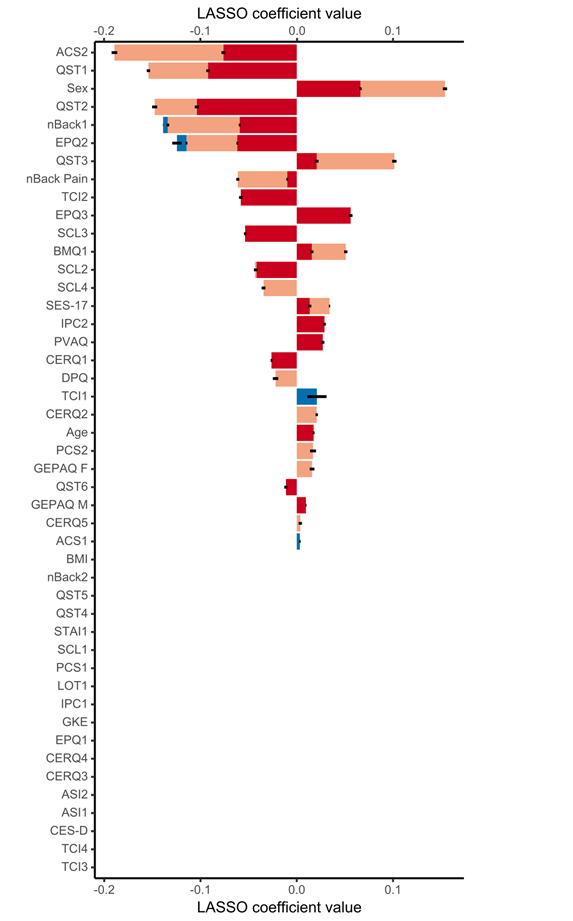


**Fig. S2**: **Complete LASSO results.** The stacked bar plots display all LASSO coefficients for each modality with standard deviations of 1000 iterations. An absent bar means that the individual characteristic was not selected through LASSO and therefore has no coefficient. Individual characteristics are ranked by summed overall coefficients to facilitate comparison with Fig S3. Individual characteristics are termed by the name of the principal component that consists of the abbreviation of the assessment and the number of the component.

*
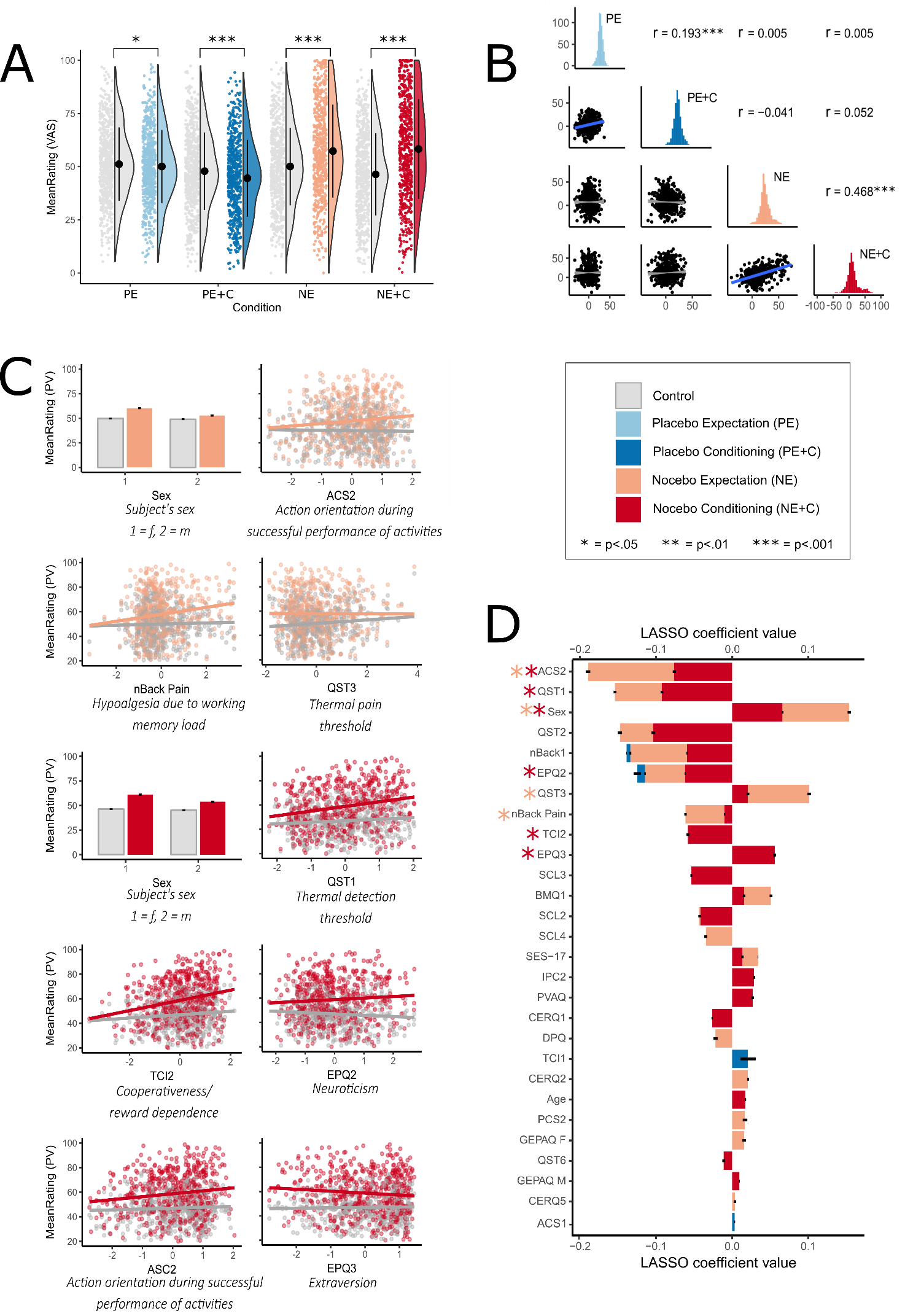
*
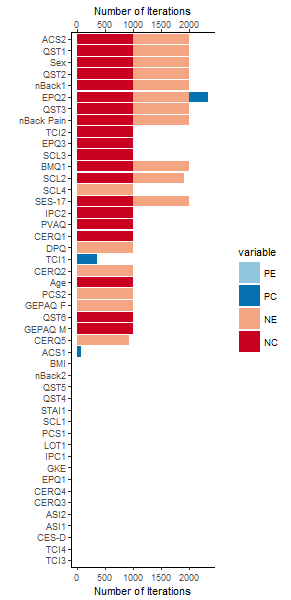


**Fig. S3: LASSO selection rates.**. The stacked bar plots display the selection rates based on 1000 iteration of LASSO. An absent individual characteristic means that the variable was not selected by LASSO. Individual characteristics are ranked by summed overall coefficients to facilitate comparison with Fig S1. Individual characteristics are termed by the name of the principal component that consists of the abbreviation of the assessment and the number of the component.

*
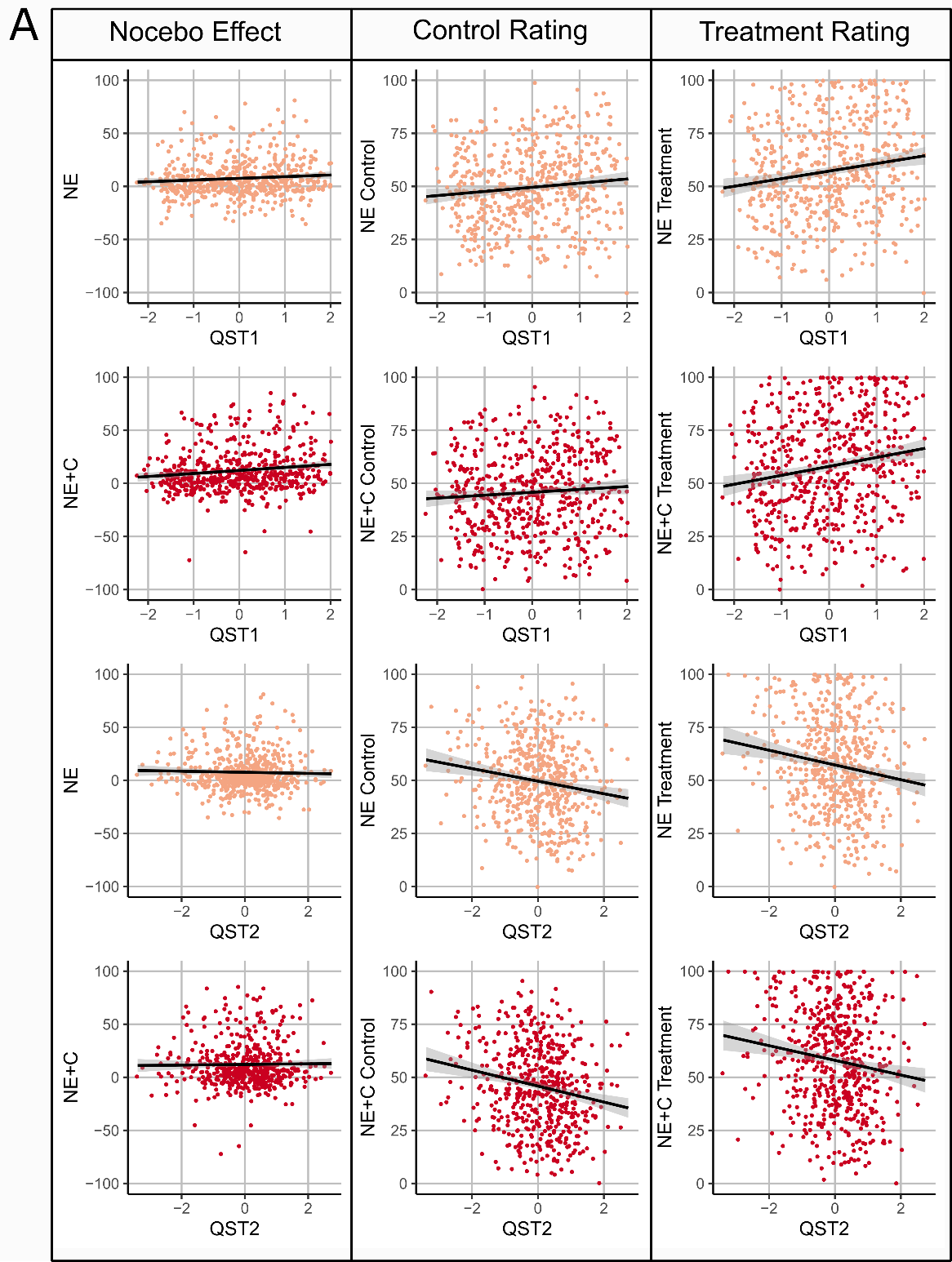
*

**Fig. S4 A-E: Illustration of prediction of nocebo effect for the ten best predictors.** Plots show effect of predictors on rating differences (left column), control ratings (middle column) and treatment ratings (right column) for nocebo expectation responses (NE, odd rows) and nocebo expectation plus conditioning responses (NE+C, even rows). The y-axis displays the individual nocebo response whereas the x-axis displays the individual score on the principal component (e.g. QST1, Thermal Pain Threshold).


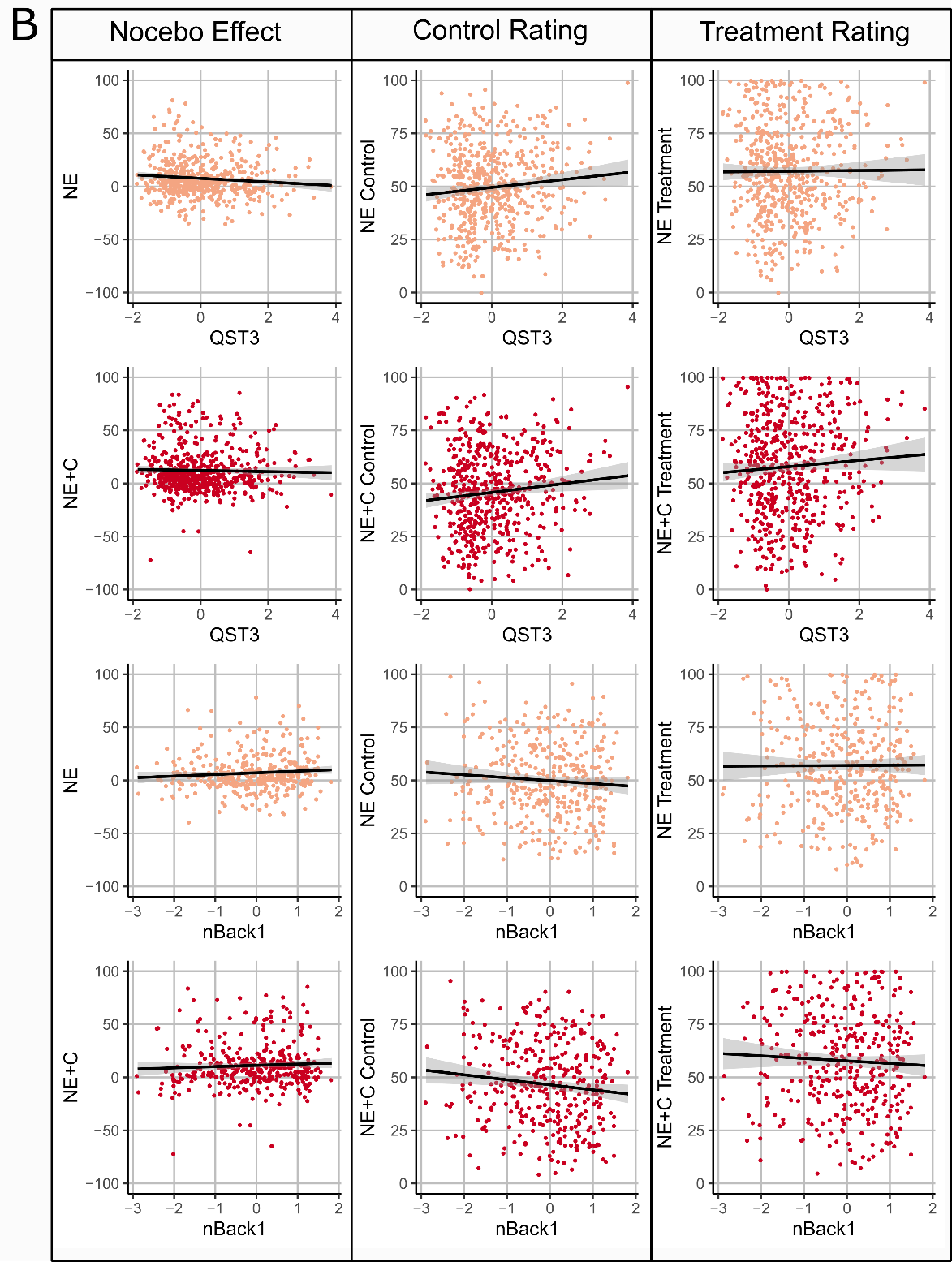


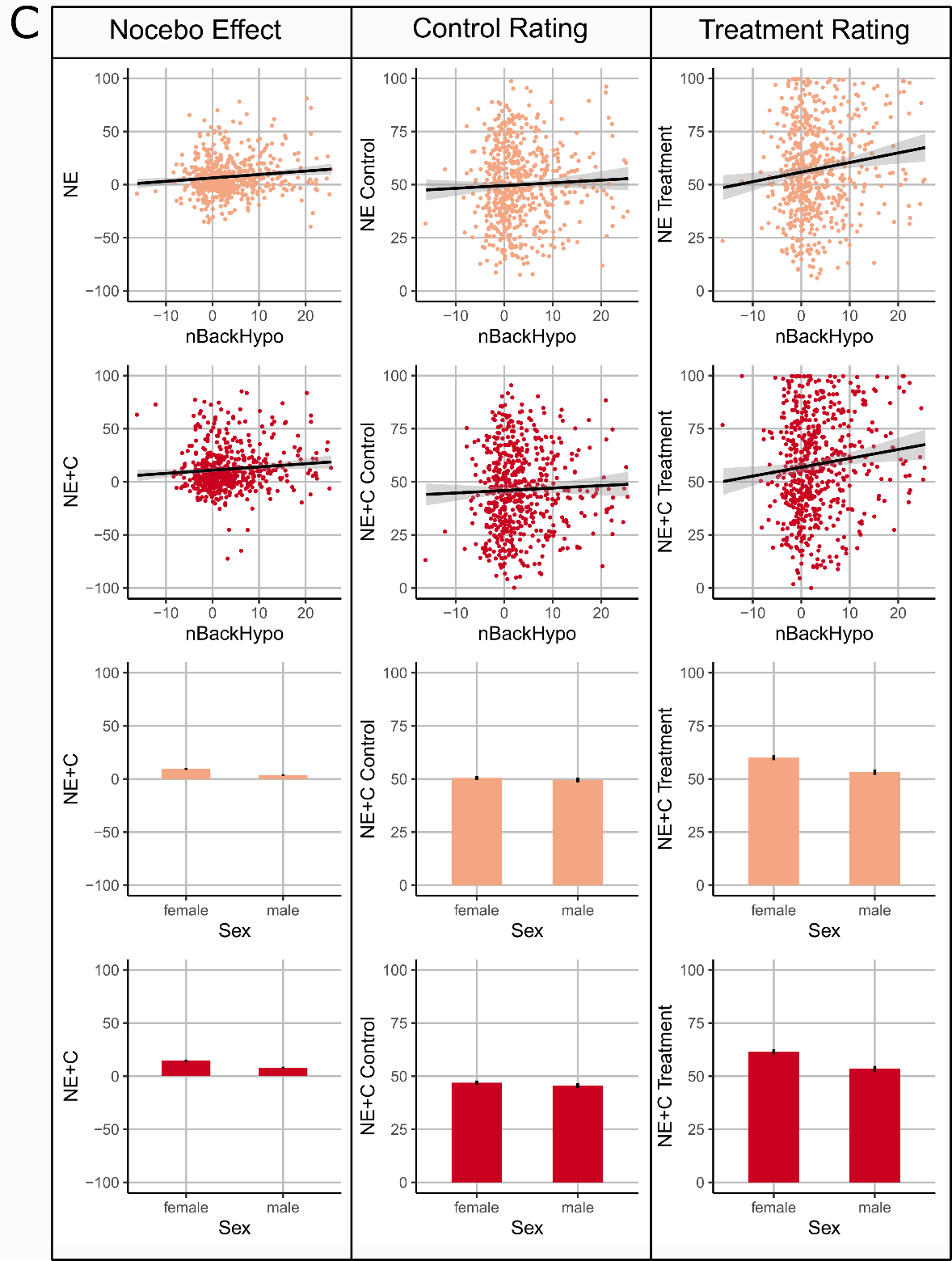


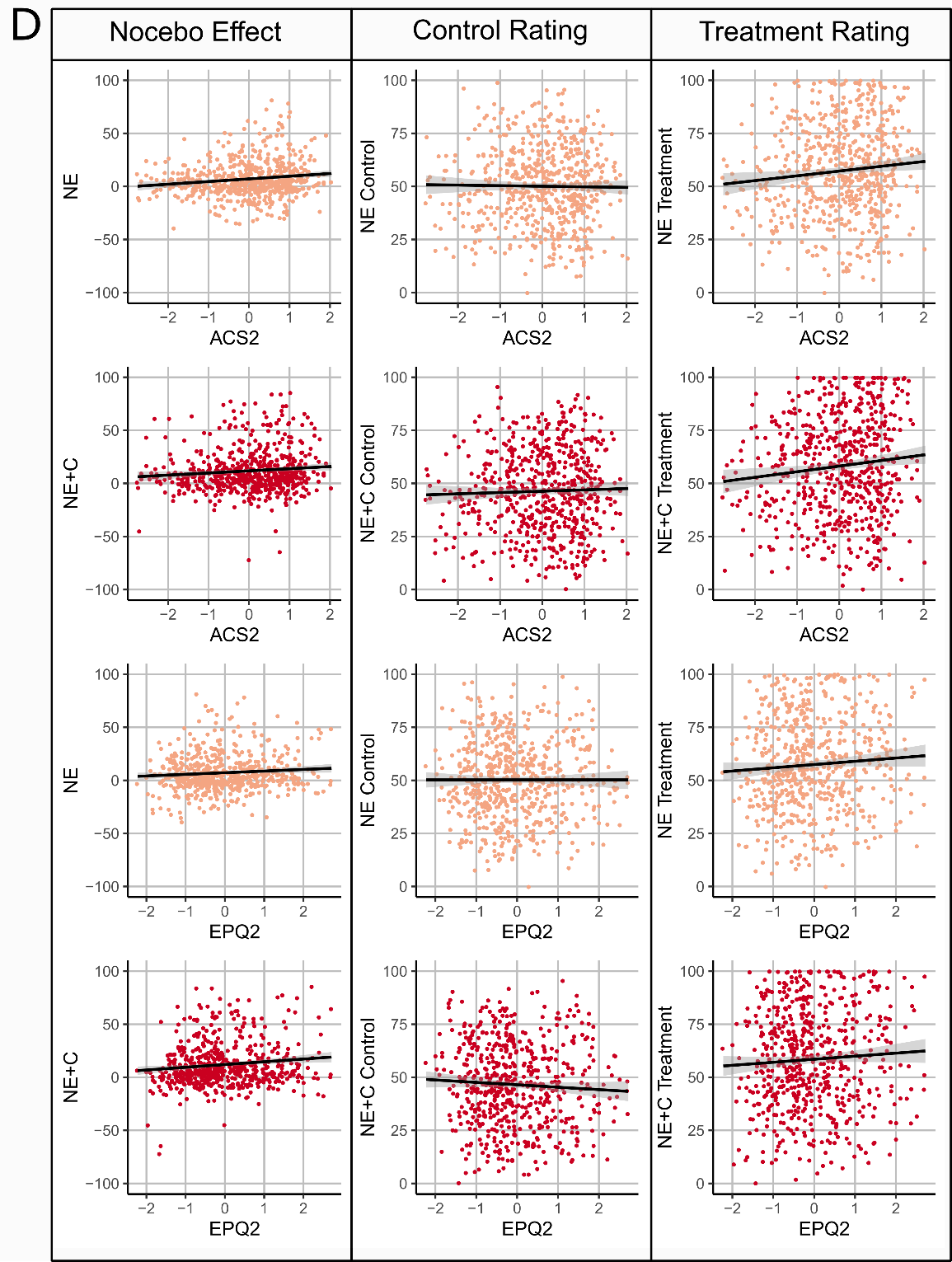


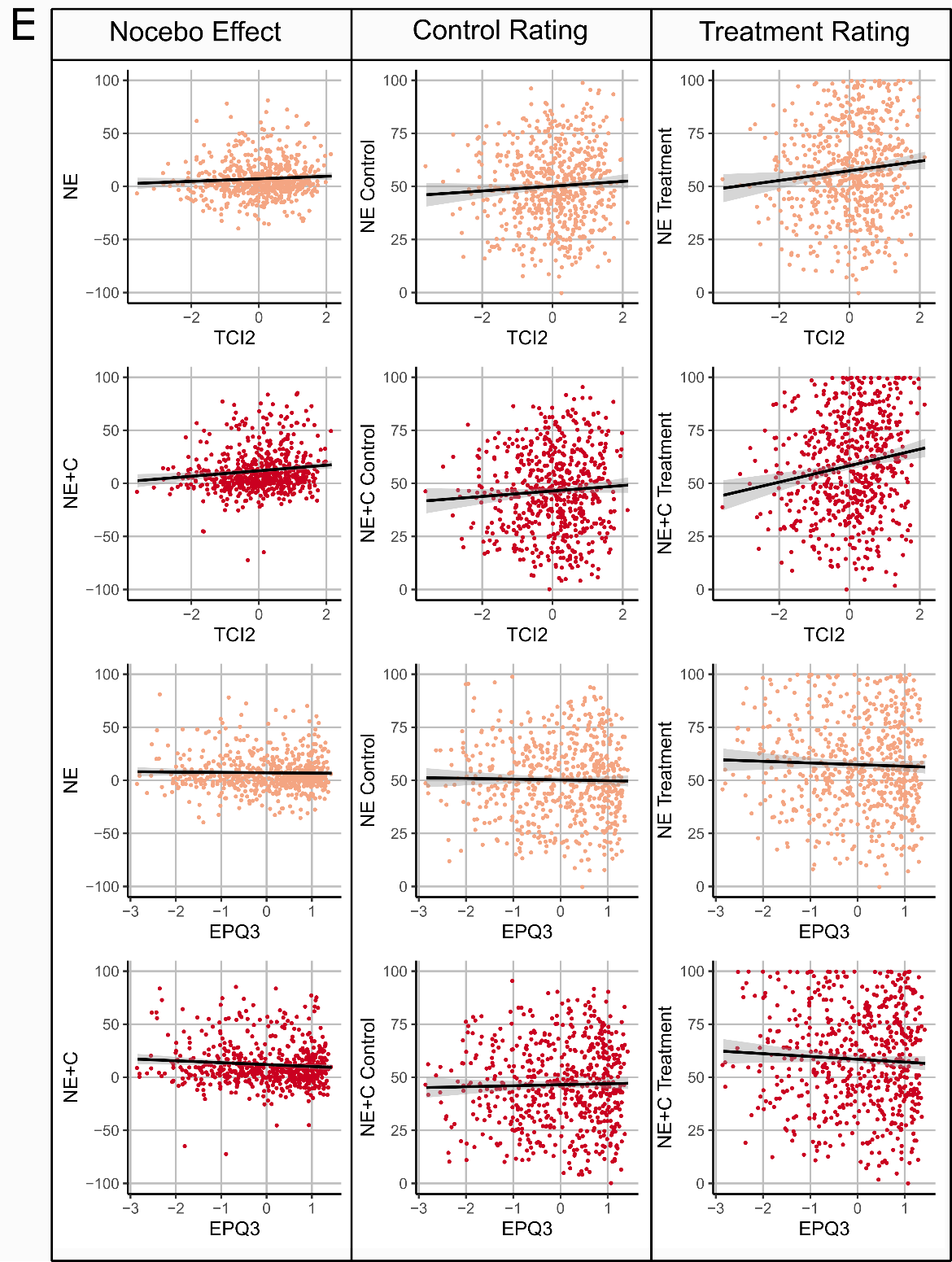

**Fig. S5: Thermoception thresholds predict nocebo effects: results and further hypothesis**. **(A)** Thermoception ranges in high nocebo responders and low nocebo responders. The box plot illustrates the distribution of warm thermoception ranges (heat pain threshold (HPT) minus warmth detection threshold (WDT)) with subjacent raw data points for a between-group comparison. Low nocebo responders (according to the lower quartile of nocebo conditioning responses, n = 149, cyan) show smaller thermoception ranges compared to high nocebo responders (according to the upper quartile of nocebo conditioning responses, n = 149, purple). **(B)** Median range of thermoception for low and high nocebo responders. Median warmth detection thresholds and median heat pain thresholds were calculated for low nocebo responder and high nocebo responder. As illustrated, low nocebo responders show a smaller range (mean WDT 33.7, mean HPT 42.0) whereas high nocebo responders show a larger range (mean WDT 33.7, mean HPT 43.5). A larger range in thermoception might indicate a lower precision whereas a smaller thermoception might indicate higher precision. **(C)** Lower thermoception precision predicting higher nocebo effects. Illustration of the hypothesized relationship of thermoception precision and nocebo effects based on a Bayesian integration framework. The observation (likelihood) in purple is centered on VAS 60, which reflects the actual stimulus, whereas the expectancy (prior) in red is centered on VAS 80, which is congruent with the stimuli of the conditioning phase. The sensory input has a wider spread compared to the expectancy and therefore less impact on the resulting pain rating (posterior) in red. This imbalance in impact is illustrated in the adjacent scales in which the observation with low precision is weighing less than the expectancy. **(D)** Higher thermoception ranges predicting lower nocebo effects. In comparison with (C), expectancy (in red) is still centered on VAS 80 and has the same spread, whereas the observation (in cyan) is still centered on VAS 60 but has a smaller spread and therefore higher precision. This leads to a higher impact on the rating of the observation compared to the expectancy and consequently to a smaller nocebo effect. Note that (C) and (D) are only illustrative examples.


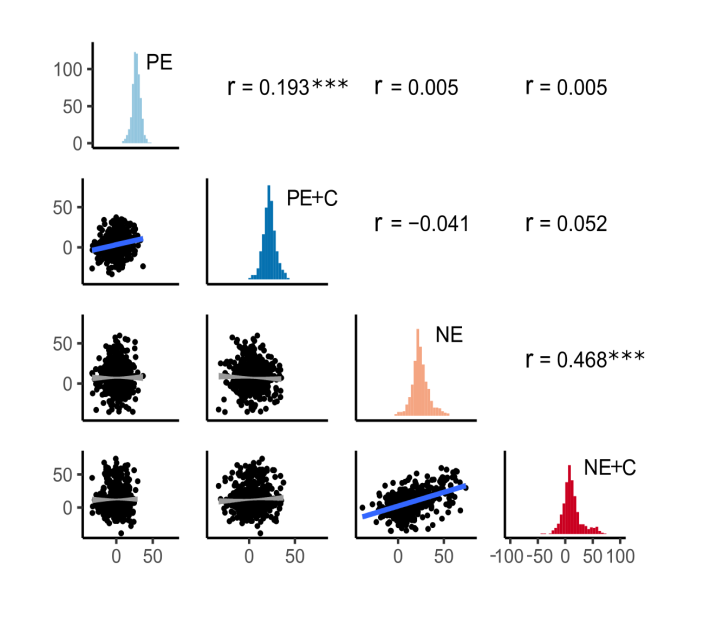


**Fig. S6: Correlation analysis of placebo and nocebo effects.** The diagonal displays histograms. Above diagonal: Correlation coefficients (*** = p < 0.001). Below diagonal: Scatterplots with blue regression line. The figure is color-coded for: Placebo Expectation (PE) in light blue, Placebo Conditioning (PE+C) in dark blue, Nocebo Expectation (NE) in orange, Nocebo Conditioning (NE+C) in red.


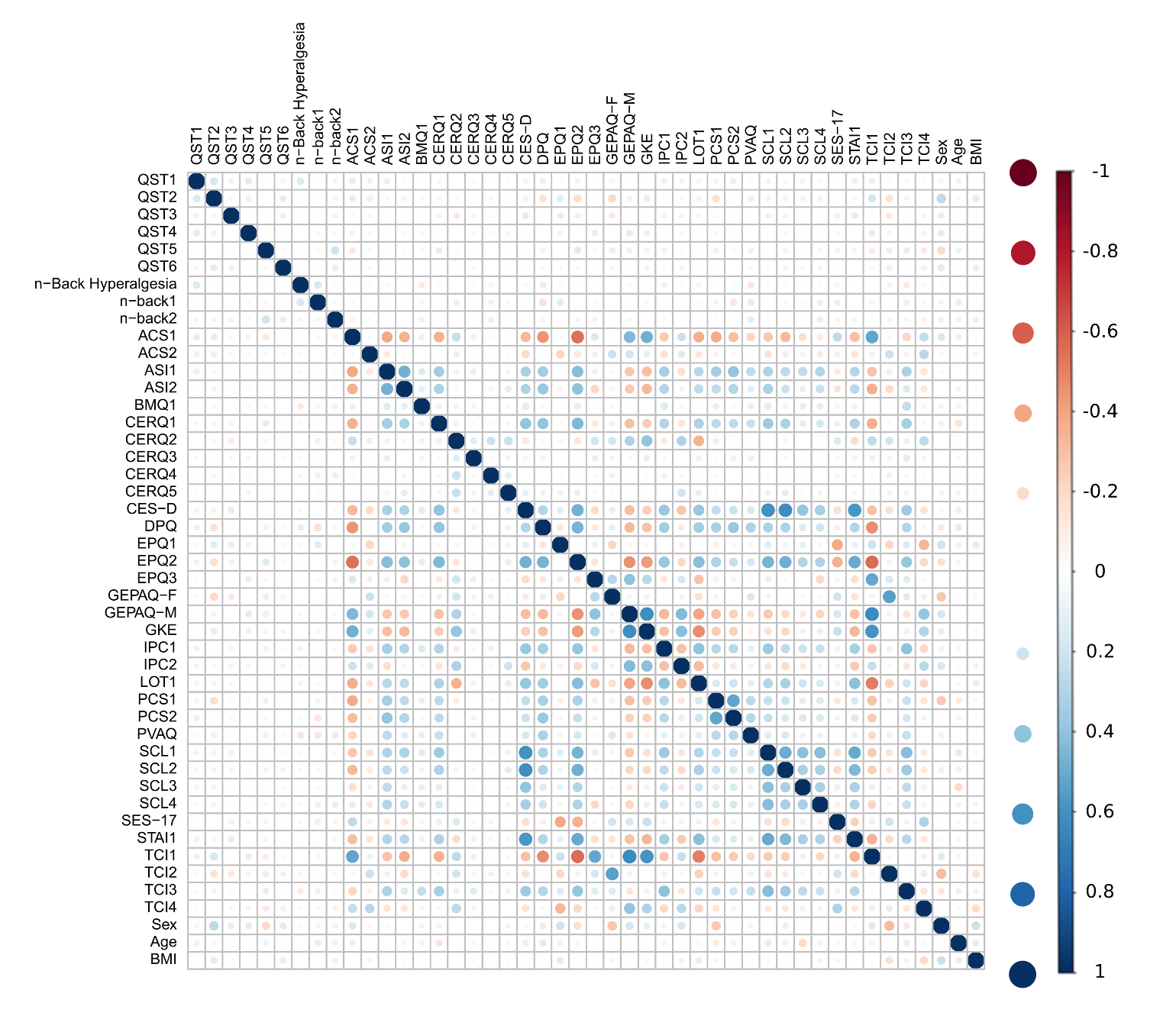


**Fig. S7: Intercorrelation between all principal components.** Circle size reflects correlation coefficient. Blue circles indicate positive coefficients and red circles indicate negative coefficients as indicated in the legend on the right.

# Table S1: Complete LASSO Results

**Table S1: This table provides the least absolute selection and shrinkage operator (LASSO) results.** For each individual characteristic, the coefficient (Coef) and standard deviation (SD) of 1000 iterations are displayed. The individual characteristics are termed by the name of the principal component that consists of the abbreviation of the assessment and the number of the component. Results are reported for all modalities: Placebo Expectation (PE), Placebo Expectation plus Conditioning (PE+C), Nocebo Expectation (NE) and Nocebo Expectation plus Conditioning (NE+C). Empty cells indicate that the individual characteristic was not selected through LASSO and has therefore no coefficient or standard deviation to report. The individual characteristics are ordered by summed coefficients over all modalities.

| **Variable** | **Coef PE** | **SD PE** | **Coef PE+C** | **SD PE+C** | **Coef NE** | **SD NE** | **Coef NE+C** | **SD NE+C** |
| --- | --- | --- | --- | --- | --- | --- | --- | --- |
| **ACS2** | - | - | - | - | -0.1133 | 0.0029 | -0.0762 | 0.0021 |
| **QST1** | - | - | - | - | -0.0619 | 0.0017 | -0.0923 | 0.0018 |
| **Sex** | - | - | - | - | 0.0878 | 0.0022 | 0.0662 | 0.0013 |
| **QST2** | - | - | - | - | -0.0438 | 0.0027 | -0.1038 | 0.0022 |
| **nBack1** | - | - | -0.0049 | 0.0002 | -0.0747 | 0.0015 | -0.0593 | 0.0010 |
| **EPQ2** | - | - | -0.0100 | 0.0049 | -0.0528 | 0.0009 | -0.0618 | 0.0009 |
| **QST3** | - | - | - | - | 0.0806 | 0.0023 | 0.0208 | 0.0018 |
| **nBack Pain** | - | - | - | - | -0.0513 | 0.0016 | -0.0100 | 0.0009 |
| **TCI2** | - | - | - | - | - | - | -0.0582 | 0.0019 |
| **EPQ3** | - | - | - | - | - | - | 0.0560 | 0.0019 |
| **SCL3** | - | - | - | - | - | - | -0.0537 | 0.0012 |
| **BMQ1** | - | - | - | - | 0.0352 | 0.0017 | 0.0157 | 0.0015 |
| **SCL2** | - | - | - | - | -0.0018 | 0.0010 | -0.0417 | 0.0009 |
| **SCL4** | - | - | - | - | -0.0345 | 0.0020 | - | - |
| **SES-17** | - | - | - | - | 0.0205 | 0.0006 | 0.0136 | 0.0016 |
| **IPC2** | - | - | - | - | - | - | 0.0288 | 0.0014 |
| **PVAQ** | - | - | - | - | - | - | 0.0270 | 0.0017 |
| **CERQ1** | - | - | - | - | - | - | -0.0263 | 0.0009 |
| **DPQ** | - | - | - | - | -0.0220 | 0.0029 | - | - |
| **TCI1** | - | - | 0.0209 | 0.0101 | - | - | - | - |
| **CERQ2** | - | - | - | - | 0.0207 | 0.0012 | - | - |
| **Age** | - | - | - | - | - | - | 0.0175 | 0.0010 |
| **PCS2** | - | - | - | - | 0.0169 | 0.0031 | - | - |
| **GEPAQ F** | - | - | - | - | 0.0159 | 0.0025 | - | - |
| **QST6** | - | - | - | - | - | - | -0.0112 | 0.0017 |
| **GEPAQ M** | - | - | - | - | - | - | 0.0095 | 0.0006 |
| **CERQ5** | - | - | - | - | 0.0037 | 0.0017 | - | - |
| **ACS1** | - | - | 0.0032 | 0.0008 | - | - | - | - |
| **BMI** | - | - | - | - | - | - | - | - |
| **nBack2** | - | - | - | - | - | - | - | - |
| **QST5** | - | - | - | - | - | - | - | - |
| **QST4** | - | - | - | - | - | - | - | - |
| **STAI1** | - | - | - | - | - | - | - | - |
| **SCL1** | - | - | - | - | - | - | - | - |
| **PCS1** | - | - | - | - | - | - | - | - |
| **LOT1** | - | - | - | - | - | - | - | - |
| **IPC1** | - | - | - | - | - | - | - | - |
| **GKE** | - | - | - | - | - | - | - | - |
| **EPQ1** | - | - | - | - | - | - | - | - |
| **CERQ4** | - | - | - | - | - | - | - | - |
| **CERQ3** | - | - | - | - | - | - | - | - |
| **ASI2** | - | - | - | - | - | - | - | - |
| **ASI1** | - | - | - | - | - | - | - | - |
| **CES-D** | - | - | - | - | - | - | - | - |
| **TCI4** | - | - | - | - | - | - | - | - |
| **TCI3** | - | - | - | - | - | - | - | - |

# Table S2: Exclusion criteria

**Table S2: Exclusion criteria.** Number of participants excluded by criterion, sum per each predictor group and sum overall

| **QST** |  |  |  |
| --- | --- | --- | --- |
| 11 variables |  |  |  |
| CDT, WDT, CPT, HPT, MDT, MPT, MPS, ALL, WUR, VDT, PPT |  |  |  |
| **Criterion description** | **n** | **Sum** | **Sum overall** |
| 3 or more outlier in 11 QST variables (mean+3SD) | 11 | **11** | **11** |
| Mean deviation of 11 QST variables too high/low (mean±3SD) | 7 | **13** | **13** |
|  |  |  |  |
| **QUESTIONNAIRES** |  |  |  |
| 67 questionnaire variables |  |  |  |
| 703 questions in total |  |  |  |
| **Criterion description** | **n** | **Sum** | **Sum overall** |
| Same answer too many times (mean+3SD = 80) | 15 | **15** | **28** |
| Changed the answer too many times (mean+3SD = 478) | 6 | **21** | **34** |
| Changed the answer not enough (mean+3SD = 271) | 2 | **21** | **34** |
| Too low intercorrelation on the highest positively intercorrelated answers (2x30 items) (mean+3SD) | 4 | **25** | **38** |
| Answered "Yes" to "Did you lie very often in this questionnaire?" | 4 | **30** | **42** |
| 5 or more outlier in 67 questionnaire variables (mean+3SD) | 14 | **44** | **56** |
| Mean deviation of 67 questionnaire variables too high/low (mean±3SD) | 11 | **44** | **56** |
|  |  |  |  |
| **N-Back** |  |  |  |
| 21 variables |  |  |  |
| For 1-back, 2-back and the Difference: VAS, Hits, False Alarms, Errors, dPrime, RT Hits, RT False Alarms | | |  |
| **Criterion description** | **n** | **Sum** | **Sum overall** |
| Error rate in 1back above mean+1SD | 46 | **46** | **94** |
| 5 or more outlier in 21 n-back variables (mean+3SD) | 20 | **49** | **96** |
| Mean deviation of 21 n-back variables too high/low (mean±3SD) | 16 | **49** | **96** |

# Table S3: Participant characteristics and questionnaires

**Table S3: Participant characteristics for the complete sample (N = 720).** Mean value and standard deviation of placebo and nocebo effects, questionnaires, quantitative sensory tests and working memory task (n-Back).

|  |  | **Mean** | **SD** |
| --- | --- | --- | --- |
|  | **Sociodemographics** |  |  |
| Age |  | 24.58 | 3.58 |
| % Female | | 59.45 |  |
| BMI |  | 22.85 | 3.49 |
|  |  |  |  |
|  | **Placebo and Nocebo Effects** |  |  |
| Placebo Expectation | | 1.06 | 14.37 |
| Placebo Expectation plus Conditioning | | 3.70 | 14.36 |
| Nocebo Expectation | | 8.47 | 20.54 |
| Nocebo Expectation plus Conditioning | | 13.64 | 23.44 |
|  | |  | |
|  | **Action Control Scale (ACS)** |  |  |
| Action orientation subsequent to failure vs. Preoccupation | | 5.07 | 2.85 |
| Prospective and decision-related action orientation vs. Hesitation | | 4.85 | 3.03 |
| Action orientation during (successful) performance of activities (intrinsic orientation) vs. volatility | | 9.08 | 2.19 |
|  |  |  |  |
|  | **Anxiety-Sensitivity Index-3 (ASI-3)** |  |  |
| Social Concerns | | 11.19 | 4.12 |
| Physical Concerns | | 15.85 | 4.62 |
| Cognitive Concerns | | 10.81 | 3.73 |
|  |  |  |  |
|  | **Beliefs about Medicine Questionnaire (BMQ)** |  |  |
| General Overuse | | 14.48 | 2.93 |
| General Harm | | 9.13 | 2.90 |
|  |  |  |  |
|  | **Cognitive Emotion Regulation Questionnaire (CERQ)** |  |  |
| Self-Blame | | 5.59 | 1.88 |
| Acceptance | | 7.02 | 2.03 |
| Rumination | | 5.62 | 1.78 |
| Positive Refocusing | | 4.88 | 1.85 |
| Refocus on Planning | | 7.31 | 1.81 |
| Positive Reappraisal | | 7.62 | 1.87 |
| Putting into Perspective | | 6.83 | 1.91 |
| Catastrophizing | | 4.05 | 1.80 |
| Blaming Others | | 3.59 | 1.17 |
|  |  |  |  |
|  | **Center for Epidemiologic Studies Depression Scale (CES-D)** |  |  |
| Depression Index | | 6.70 | 5.04 |
|  |  |  |  |
|  | **Defensive Pessimism Questionnaire (DPQ)** |  |  |
| Defensive Pessimism Score | | 46.65 | 12.01 |
|  |  |  |  |
|  | **Eysenck Personality Questionnaire (EPQ)** |  |  |
| Psychoticism | | 2.86 | 1.93 |
| Extraversion | | 8.32 | 3.19 |
| Neuroticism | | 3.71 | 2.84 |
| Control Scale | | 2.42 | 2.02 |
|  |  |  |  |
|  | **German Extended Personal Attributes Questionnaire (GEPAQ)** |  |  |
| Masculinity (Instrumentality) | | 3.54 | 0.56 |
| Femininity (Expressivity) | | 3.99 | 0.50 |
|  | |  |  |
|  |  | **Mean** | **SD** |
|  | **General Competence Expectancy Test (GKE)** |  |  |
| Self-Efficacy | | 3.07 | 0.39 |
|  |  |  |  |
|  | **Internality, Powerful Others and Chance Scale (IPC)** |  |  |
| Internal | | 36.85 | 3.87 |
| Powerful Others | | 24.36 | 4.55 |
| Chance | | 24.68 | 4.50 |
|  |  |  |  |
|  | **Life-Orientation-Test (LOT)** |  |  |
| Pessimism Score | | 3.79 | 2.29 |
| Optimism Score | | 3.30 | 2.44 |
|  |  |  |  |
|  | **Pain Catastrophizing Scale (PCS)** |  |  |
| Magnification | | 1.39 | 0.79 |
| Rumination | | 2.22 | 0.97 |
| Helplessness | | 1.27 | 0.75 |
|  |  |  |  |
|  | **Pain Vigilance and Awareness Questionnaire (PVAQ)** |  |  |
| Pain Vigilance Awareness Score | | 38.12 | 9.68 |
|  |  |  |  |
|  | **Symptom Checklist-90 (SCL-90)** |  |  |
| Somatization | | 0.31 | 0.26 |
| Obsessive-Compulsive | | 0.57 | 0.41 |
| Interpersonal Sensitivity | | 0.43 | 0.40 |
| Depression | | 0.55 | 0.44 |
| Anxiety | | 0.28 | 0.25 |
| Hostility | | 0.34 | 0.35 |
| Phobic Anxiety | | 0.11 | 0.18 |
| Paranoid Ideation | | 0.33 | 0.38 |
| Psychoticism | | 0.18 | 0.24 |
|  |  |  |  |
|  | **Social Desireability Scale-17 (SES-17)** |  |  |
| Social Desirability Score | | 9.74 | 3.16 |
|  |  |  |  |
|  | **State-Trait-Anxiety Inventore (STAI)** |  |  |
| State Anxiety | | 35.53 | 7.33 |
| Trait Anxiety | | 36.81 | 8.05 |
|  |  |  |  |
|  | **Temperament Character Inventory (TCI)** |  |  |
| Novelty Seeking | | 5.56 | 1.40 |
| Harm Avoidance | | 3.34 | 1.63 |
| Reward Dependence | | 5.34 | 1.24 |
| Persistence | | 4.46 | 2.11 |
| Self-Directedness | | 6.43 | 1.34 |
| Cooperativeness | | 6.54 | 1.06 |
| Self-Transcendence | | 3.54 | 1.97 |
| Social Desirability | | 6.44 | 1.86 |

|  |  | **Mean** | **SD** |
| --- | --- | --- | --- |
|  | **Quantitative Sensory Testing (QST)** |  |  |
| Cold Detection Threshold | | 30.72 | 0.73 |
| Warmth Detection Threshold | | 33.90 | 0.75 |
| Cold Pain Threshold | | 15.98 | 10.09 |
| Heat Pain Threshold | | 42.03 | 3.64 |
| Mechanical Detection Threshold | | 3.43 | 2.75 |
| Mechanical Pain Threshold | | 87.89 | 90.57 |
| Dynamic Mechanical Allodynia | | 0.03 | 0.42 |
| Mechanical Pain Sensitivity | | 4.03 | 4.56 |
| Windup Ratio (256mN) | | 2.53 | 4.46 |
| Vibration Detection Threshold | | 7.23 | 0.53 |
| Pressure Pain Threshold | | 4.69 | 1.74 |
|  |  |  |  |
|  | **Working Memory Task (n-back)** |  |  |
| VAS Difference 1-back - 2-back | | 3.59 | 6.80 |
| Hits Difference 1-back - 2-back | | 0.15 | 0.11 |
| False Alarms Difference 1-back - 2-back | | -0.05 | 0.04 |
| Errors Difference 1-back - 2-back | | -0.20 | 0.13 |
| Reaction Times Hits Difference 1-back - 2-back | | -111.60 | 77.52 |
| Reaction Times False Alarms Difference 1-back - 2-back | | -121.51 | 196.02 |

# Table S4: Verbal Instructions Placebo and Nocebo Ointment

**Table S4: Verbal instructions Placebo and Nocebo Ointment.** This table displays the exact verbal instructions given by the experimenter to the participant in German (original) and English (translated).

| **Treatment** | **Verbal Instructions** |
| --- | --- |
| Placebo Ointment | German: *“Dies ist die Emla-Salbe. Sie enthält den Wirkstoff Lidocaine, welcher als schnell wirkendes Lokalanästhetikum benutzt wird. Es wird z.B beim Zahnarzt in Form von Injektionen verabreicht. In Form von einer Salbe ist der Wirkstoff geringer dosiert und wird z.B. zur Schmerzausschaltung beim Blutabnehmen bei Kindern verwendet. Lidocain diffundiert durch die Haut und hemmt die Natriumkanäle, so dass die Reizweiterleitung des Gewebes reduziert wird und so das Schmerzempfinden der Stelle herabgesetzt wird. Es wird sowohl das Empfinden von Druck und Schmerz als auch von Wärme und Kälte reduziert. Die Emla-Salbe hat nachgewiesenermaßen in zahlreichen Studien zu einer Schmerzreduktion geführt. 9 von 10 Menschen zeigen eine Schmerzreduktion. Die Emla-Salbe ist in der Apotheke rezeptfrei zu erhalten, wir testen demnach kein neues Präparat, sondern nutzen lediglich ein bereits bestehendes Pharmaprodukt um die Schmerzwahrnehmung zu verändern und so genauer zu untersuchen. Zusätzlich werde ich eine Kontrollsalbe auf eine andere Stelle auftragen, diese ist unarzneilich und enthält keinen Wirkstoff. Ich decke die Stellen mit Pflastern ab, damit Sie sich trotz der Salbe frei bewegen können und die Salbe gut einwirken kann.“*  English translation: *“This is the Emla ointment. It contains the active ingredient lidocaine, which is used as a fast-acting local anesthetic. It is administered e.g. at the dentist in the form of injections. In the form of an ointment, the active ingredient is in a lower dosage and is used, for example, to eliminate pain when taking blood samples from children. Lidocaine diffuses through the skin and inhibits the sodium channels so that the transmission of stimuli to the tissue is reduced and the sensation of pain in the area is decreased. It reduces the sensation of pressure and pain as well as heat and cold. The Emla Ointment is available in pharmacies without a prescription, so we are not testing a new drug, but only using an existing pharmaceutical product to change the perception of pain and thus to investigate it more precisely. In addition, I will apply a control ointment to another area, this one is not medicinal and contains no active ingredient. The areas will be covered with plasters so that you can move freely despite the ointment and the ointment can work well.”* |
| Nocebo Ointment | German: *“Dies ist die ABC-Salbe, teilweise vielleicht eher bekannt von den ABC-Pflastern. Die ABC-Salbe enthält den Wirkstoff Capsaicin, welcher aus der Chilischote gewonnen wird. Capsaicin reizen die Nervenenden von Schmerzrezeptoren/Nozirezeptor, dadurch entsteht Hitze welcher im therapeutischen Setting zu einer Entspannung führen soll. Die Salbe wird vor allem bei Muskelschmerzen im Bereich der Wirbelsäule und zur Rheumatherapie angewendet. Hier im Experiment modulieren wir durch die ABC-Salbe Ihr Hitzeschmerzempfinden, da Sie durch die Salbe die Hitzereize verstärkt spüren. Während der Einwirkzeit kann es vorkommen, dass es ein wenig kribbelt, sobald es aber brennt oder wehtut, sollten Sie mir direkt Bescheid sagen.”*  English translation: *“This is the ABC ointment, perhaps better known from the ABC plasters. The ABC ointment contains the active ingredient capsaicin, which is extracted from chili pepper. Capsaicin irritates the nerve endings of pain receptors/nociceptors, thereby producing heat which leads to relaxation in a therapeutic setting. The ointment is mainly used for muscle pain in the spine area and for rheumatism therapy. Here in the experiment we modulate your heat pain sensation by the ABC ointment because you will feel the heat stimulus strengthened by the ointment. During the time of application, it can happen that it tingles a little, but as soon as it burns or hurts, you should let me know directly.”* |

# Table S5: Questionnaires

**Table S5: List of all questionnaires used in the study with references.**

| **Questionnaire** | **Reference** |
| --- | --- |
| Action Control Scale 90 (ACS-90) | Kuhl, J. (1990). Fragebogen zur Erfassung der Handlungskontrolle: HAKEMP-90. *Universität Osnabrück*. |
| Anxiety Sensitivity-Index-3 (ASI-3) | Taylor, S., Zvolensky, M. J., Cox, B. J., Deacon, B., Heimberg, R. G., Ledley, D. R., … Stewart, S. H. (2007). Robust dimensions of anxiety sensitivity: Development and initial validation of the Anxiety Sensitivity Index-3. *Psychological Assessment*, *19*(2), 176. |
| Beliefs about Medicines Questionnaires (BMQ) | Horne, R., Weinman, J., & Hankins, M. (1999). The beliefs about medicines questionnaire: The development and evaluation of a new method for assessing the cognitive representation of medication. *Psychology & Health*, *14*(1), 1–24. |
| Center for Epidemiologic Studies Depression Scale (CES-D-Scale) | Radloff, L. S. (1977). The CES-D scale: A self-report depression scale for research in the general population. *Applied Psychological Measurement*, *1*(3), 385–401. |
| Cognitive Emotion Regulation Questionnaire (CERQ) | Garnefski, N., & Kraaij, V. (2007). The cognitive emotion regulation questionnaire. *European Journal of Psychological Assessment*, *23*(3), 141–149. |
| Defensive Pessimism Questionnaire (DPQ) | Norem, J. K., & Cantor, N. (1986). Defensive pessimism: Harnessing anxiety as motivation. *Journal of Personality and Social Psychology*, *51*(6), 1208. |
| Eysenck Personality Questionnaire (EPQ-RK) | Eysenck, S. B. G., Eysenck, H. J., & Barrett, P. (1985). A revised version of the psychoticism scale. *Personality and Individual Differences*, *6*(1), 21–29. |
| German Extended Personal Attributes Questionnaire (GEPAQ) | Runge, T. E., Frey, D., Gollwitzer, P. M., Helmreich, R. L., & Spence, J. T. (1981). Masculine (instrumental) and feminine (expressive) traits: A comparison between students in the United States and West Germany. *Journal of Cross-Cultural Psychology*, *12*(2), 142–162. |
| General Competence Expectancy Test (GKE) | Schwarzer, R., & Jerusalem, M. (1995). Generalized self-efficacy scale. In *Measures in health psychology: A user’s portfolio. Causal and control beliefs* (Vol. 1, pp. 35–37). Windsor, UK: NFER-NELSON. |
| Internality, Powerful Other and Chance Scale (IPC) | Rotter, J. B. (1966). Generalized expectancies for internal versus external control of reinforcement. *Psychological Monographs: General and Applied*, *80*(1). |
| Life-Orientation-Test (LOT) | Scheier, M. F., & Carver, C. S. (1985). *Optimism, Coping, and Health: Assessment and Implications of Generalized Outcome Expectancies*. *4*(3), 219–247. |
| Pain Catastrophizing Scale (PCS) | Sullivan, M. J. L. (2009). *The Pain Catastrophizing Scale*. Montreal, Quebec: McGill University. |
| Pain Vigilance and Awareness Questionnaire (PVAQ) | McCracken, L. M. (1997). “Attention” to pain in persons with chronic pain: A behavioral approach. *Behavior Therapy*, *28*(2), 271–284. |
| Symptom Checklist 90 (SCL-90) | Derogatis, L. R., & Unger, R. (2010). Symptom checklist‐90‐revised. *The Corsini Encyclopedia of Psychology*, 1–2. |
| Social Desirability Scale-17 (SDS-17) | Stöber, J. (1999). Die Soziale-Erwünschtheits-Skala-17 (SES-17): Entwicklung und erste Befunde zu Reliabilität und Validität. *Diagnostica*, *45*(4), 173–177. |
| State Trait Anxiety Inventory (STAI) | Spielberger, C. D. (1983). *State-trait anxiety inventory for adults*. Palo Alto, CA: Consulting Psychologists Press. |
| Temperament Character Inventory (TCI) | Cloninger, C. R. (Ed.). (1994). *The temperament and character inventory (TCI): A guide to its development and use* (1st ed). St. Louis, Mo: Center for Psychobiology of Personality, Washington University. |
